# Tocilizumab in patients admitted to hospital with COVID-19 (RECOVERY): a randomised, controlled, open-label, platform trial

**DOI:** 10.1016/S0140-6736(21)00676-0

**Published:** 2021-05-01

**Authors:** RECOVERY Collaborative Group, Obbina Abani, Ali Abbas, Fatima Abbas, Mustafa Abbas, Sadia Abbasi, Hakam Abbass, Alfie Abbott, Nabeel Abdallah, Ashraf Abdelaziz, Mohamed Abdelfattah, Bushra Abdelqader, Basir Abdul, Althaf Abdul Rasheed, Ajibode Abdulakeem, Rezan Abdul-Kadir, Abdulfatahi Abdulmumeen, Rasheed Abdul-Raheem, Niyaz Abdulshukkoor, Kula Abdusamad, Yazeed Abed El Khaleq, Mai Abedalla, Abeer Abeer Ul Amna, Katrina Abernethy, Adebanke Aboaba, Hani Abo-Leyah, Ahmed Abou-Haggar, Mahmoud Abouibrahim, Miriam Abraham, Tizzy Abraham, Abraheem Abraheem, Judith Abrams, Hyacinth-John Abu, Ahmed Abu-Arafeh, Syed M Abubacker, Akata Abung, Yaa Aceampong, Amaka Achara, Devikumar Acharya, Sarah Acheampong, Janet Acheson, Andres Acosta, Catherine Acton, Jacqueline Adabie-Ankrah, Fiona Adam, Matthew Adam, Huzaifa Adamali, Carol Adams, Charlotte Adams, Kate Adams, Richard Adams, Tim Adams, Kirsty Adcock, Jemaimah Addai, Ade Adebiyi, Ken Adegoke, Vicki Adell, Sherna Adenwalla, Oluwasegun A Adesemoye, Emmanuel O Adewunmi, Joyce Adeyemi, Rina Adhikary, Gabrielle Adkins, Adnan Adnan, John Aeron-Thomas, Debbie Affleck, Carmel Afnan, Muhammad Afridi, Zainab A Aftab, Meenakshi Agarwal, Rachel Agbeko, Chris Agbo, Penny Agent, Sunil Aggarwal, Arameh Aghababaie, Shafana Ahamed Sadiq, Mohamed H Ahammed Nazeer, Mohammad Ahmad, Syed Ahmad, Asim Ahmed, Bilal Ahmed, Forizuddin Ahmed, Hamze Ahmed, Iram Ahmed, Irshad Ahmed, Khaled Ahmed, Liban Ahmed, Mahin Ahmed, Maria C Ahmed, Muhammad S Ahmed, Naseer Ahmed, Nausheen Ahmed, Osama Ahmed, Rajia A Ahmed, Rizwan Ahmed, Saif Ahmed, Sammiya Ahmed, Sara Ahmed, Syed Ahmed, Syed Haris Ahmed, Roa Ahmed Ali, Sana Ahmed, Sana Ahmer, Dhiraj Ail, Mark Ainsworth, Myriam Aissa, Lindianne Aitken, Bini Ajay, Abdulakeem Ajibode, Ayesha Ajmi, Nasim Akhtar, Nauman Akhtar, Suha Akili, Oludoyinsola Akindolie, Yinka Akinfenwa, Olugbenga Akinkugbe, Olugbenro Aktinade, Ahmad Al Aaraj, Asma Al Balushi, Majd Al Dakhola, Aladdin Al Swaifi, Eslam Al-Abadi, Narendra Aladangady, Ayaz Alam, Sajid Alam, Abbas Al-Asadi, Kyriaki Alatzoglou, Paul Albert, Lorraine Albon, Gemma Alcorn, Stephen Alcorn, Aggie Aldana, David Alderdice, Rayan Aldouri, Jonathan Aldridge, Nicolas Aldridge, Ana Alegria, Alison Alexander, John Alexander, Peter D G Alexander, Charlotte Alford, Julyan Al-Fori, Laith Alghazawi, Bahij Al-Hakim, Shams Al-Hity, Ali Ali, Asad Ali, Fawzia R Ali, Jawad Ali, Mariam Ali, Mohammad Ali, Nayab Ali, Oudai Ali, Sakina Ali, Syed Ali, Abid Alina, Fine Aliyuda, Katrin Alizaedeh, Maithem Al-Jibury, Saba Al-Juboori, Majid Al-Khalil, Moutaz Alkhusheh, Fiona Allan, Alison Allanson, Robert Allcock, Eireann Allen, Kerry Allen, Louise Allen, Poppy Allen, Rebecca Allen, Sam Allen, Sharon Allen, Simon Allen, Kathryn Allison, Bethan Allman, Lynne Allsop, Hassan Al-Moasseb, Magda Al-Obaidi, Lina Alomari, Akram Al-Rabahi, Bahar Al-Ramadhani, Zayneb Al-Saadi, Inji Alshaer, Rustam Al-Shahi Salman, Warkaq Al-Shamkhani, Bashar Al-Sheklly, Sara Altaf, Mary Alvarez, Maysaa Alzetani, Susan Amamou, Noor Amar, Sakkarai Ambalavanan, Sarah-Jayne Ambler, Robert Ambrogetti, Chris Ambrose, Amir Ameen, Ken Amenyah, Maria R Amezaga, Allison Amin, Amina Amin, Kanish Amin, Syed Amin, Tara Amin, Amjad Amjad, Neelma Amjad, Victoria Amosun, Khaled Amsha, Pugh Amy, Atul Anand, Samantha Anandappa, Julie Anderson, Laura Anderson, Michelle Anderson, Nicola Anderson, Rachel Anderson, Rory Anderson, Wendy Anderson, Prematie Andreou, Angela Andrews, Antonette Andrews, Jill Andrews, Kanayochukwu Aneke, Andrew Ang, Wan Wei Ang, Tammy Angel, Aramburo Angela, Paola Angelini, Lazarus Anguvaa, Oleg Anichtchik, Millicent Anim-Somuah, Krishnan Aniruddhan, Marie Anne Ledingham, Jessica Annett, Patrick James Anstey, Rebekah Anstey, Alpha Anthony, Aaron Anthony-Pillai, Philip Antill, Zhelyazkova Antonina, Varghese Anu, Muhammad Anwar, Aristeidis Apostolopoulos, Diane Appleyard, Maia Far Aquino, Bianca Araba, Samuel Aransiola, Mariana Araujo, Ann Archer, Denise Archer, Simon Archer, Christian Ardley, Ana-Maria Arias, Ryoki Arimoto, Charlotte Arkley, Charlotte Armah, Ilianna Armata, Adam Armitage, Ceri Armstrong, Maureen Armstrong, Sonia Armstrong, Philippa Armtrong, Heike Arndt, Clare Arnison-Newgass, David Arnold, Rachael Arnold, Dhawal Arora, Kavan Arora, Pardeep Arora, Rishi Arora, Arslam Arter, Ayush Arya, Rita Arya, Denisa Asandei, Adeeba Asghar, Catherine Ashbrook-Raby, Helen Ashby, Jan Ashcroft, John Ashcroft, Samuel Ashcroft, Deborah Asher, Ayesha Ashfaq, Abdul Ashish, Sally Ashman-Flavell, Sundar Ashok, Abd-El-Aziz Ashour, Muhammad Z Ashraf, Saima Ashraf, Mohammad B Ashraq, Deborah Ashton, Susan Ashton, Andrew Ashworth, Rebecca Ashworth, Arshia Aslam, Harshini Asogan, Atif Asrar, Omar Assaf, Raine Astin-Chamberlain, Deborah Athorne, Billie Atkins, Christopher Atkins, Stacey Atkins, John Atkinson, Vicki Atkinson, Brygitta Atraskiewicz, Abdul Ahmad Attia, Rita Atugonza, Rita Atugonza, Paula Aubrey, Avinash Aujayeb, Aye C T Aung, Hnin Aung, Kyaw T Aung, Yin Aung, Zaw M Aung, Emily Austin, Karen Austin, Abdusshakur Auwal, Miriam Avery, Joanne Avis, Georgina Aviss, Cristina Avram, Paula Avram, Gabriel Awadzi, Atia Awan, Aszad Aya, Eman Ayaz, Amanda Ayers, Jawwad Azam, Mohammed Azharuddin, Ghazala Aziz, N Aziz, Ali Azkoul, Ashaari Azman Shah, Giada Azzopardi, Hocine Azzoug, Fiyinfoluwa Babatunde, Melvin Babi, Babiker Babiker, Gayna Babington, Matthew Babirecki, Marta Babores, Adetona O Babs-Osibodu, Sammy Bacciarelli, Roudi Bachar, Gina Bacon, Jenny Bacon, Bibi Badal, Gurpreet R Badhan, Shreya Badhrinarayanan, Joseph P Bae, Sibel Bafekr, Alice Baggaley, Amy Baggott, Graham Bagley, Dinesh Bagmane, Lynsey Bagshaw, Kasra Bahadori, James Bailey, Katie Bailey, Lindsey Bailey, Liz Bailey, Morgan Bailey, Pippa Bailey, Sarah Bailey, Hamish Baillie, J Kenneth Baillie, Jennifer Bain, Vikram Bains, David Baird, Susan Baird, Tracy Baird, Yolanda Baird, Aiysha Bajandouh, Evelyn Baker, Johanne Baker, Josephine Baker, Kenneth Baker, Rebecca Baker, Terri-Anne Baker, Victoria Baker, Hugh Bakere, Nawar Bakerly, Michelle Baker-Moffatt, Nauman Bakhtiar, Panos Bakoulas, Niranjan Balachandran, Andrea Balan, Theodosios Balaskas, Madhu Balasubramaniam, Alison Balcombe, Alexander Baldwin, Ashley Baldwin, Caron Baldwin, Danielle Baldwin, Rebekah Baldwin-Jones, James Balfour, Ceri Ball, Kasia Ballard, Ismael Balluz, Craig Balmforth, Emese Balogh, Amir Baluwala, Gabby Bambridge, Alasdair Bamford, Amy Bamford, Peter Bamford, Adefunke Bamgboye, Elizabeth Bancroft, Hollie Bancroft, Tanya Bancroft, Joyce Banda, Krishna Bandaru, Srini Bandi, Nageswar Bandla, Somaditya Bandyopadhyam, Amit Banerjee, Ritwik Banerjee, Harrison Banks, Luke Banks, Paul Banks, Oliver Bannister, Laura Banton, Mariamma Baptist, Tanya Baqai, Ananya Mouli Baral, Desislava Baramova, Russel Barber, Emma Barbon, Monica Barbosa, Jamie Barbour, Alexander Barclay, Claire Barclay, George Bardsley, Stephanie Bareford, Shahedal Bari, Amy Barker, Debbie Barker, Helen Barker, Joseph Barker Barker, Leon Barker, Oliver Barker, Kerry Barker-Williams, Sinha Barkha, Juliana Barla, Gavin Barlow, Richard Barlow, Valerie Barlow, James Barnacle, James Barnacle, Alex Barnard, Debi Barnes, Nicky Barnes, Theresa Barnes, Calum Barnetson, Amy Barnett, Matthew Barnett, Ashton Barnett-Vanes, William Barnsley, Andrew Barr, David Barr, Shaney Barratt, Manuella Barrera, Amy Barrett, Fiona Barrett, Jessica Barrett, Jazz Bartholomew, Claire Bartlett, Georgina Bartlett, Greg Barton, Jill Barton, Lorna Barton, Rachael Barton, Rosaleen Baruah, Sonia Baryschpolec, Archana Bashyal, Betsy Basker, Ayten Basoglu, John Bassett, G Bassett, Chris Bassford, Bengisu Bassoy, Victoria Bastion, Anupam Basumatary, Tristan Bate, Harry J Bateman, Kathryn Bateman, Vhairi Bateman, Eleanor Bates, Hayley Bates, Michelle Bates, Simon Bates, Sally Batham, Ana Batista, Amit Batla, Dushyant Batra, Harry Batty, Thomas Batty, Miranda Baum, Carina Bautista, Fareha Bawa, Fatima S Bawani, Simon Bax, Matt Baxter, Nicola Baxter, Zachary Baxter, Hannah Bayes, Farid Bazari, Rohit Bazaz, Ahmad Bazli, Laura Beacham, Wendy Beadles, Philip Beak, Andy Beale, Jack Bearpark, Karen Beaumont, Dawn Beaumont-Jewell, Theresa Beaver, Sarah Beavis, Christy Beazley, Sarah Beck, Virginia Beckett, Rosie Beckitt, Heidi Beddall, Seonaid Beddows, Deborah Beeby, Gail Beech, Michelle Beecroft, Sally Beer, Jane Beety, Gabriela Bega, Alison Begg, Susan Begg, Sara Beghini, Ayesha Begum, Salman Begum, Selina Begum, Teresa Behan, Jasmine Beharry, Roya Behrouzi, Jon Beishon, Claire Beith, James Belcher, Holly Belfield, Katherine Belfield, Ajay Belgaumkar, Dina Bell, Gareth Bell, Gillian Bell, Lauren Bell, Louise Bell, Nicholas Bell, Pippa Bell, Stephanie Bell, Jennifer L Bell, Jennifer Bellamu, Mary Bellamy, Arianna Bellini, Amanda Bellis, Fionn Bellis, Lesley Bendall, Naveena Benesh, Nicola Benetti, Leonie Benham, Guy Benison-Horner, Ann Bennett, Caroline Bennett, Gillian Bennett, Kristopher Bennett, Lorraine Bennett, Sara Bennett, Karen Bennion, Vivienne Benson, Andrew Bentley, James Bentley, Ian Benton, Eva Beranova, Matthew Beresford, Colin Bergin, Malin Bergstrom, Jolanta Bernatoniene, Thomas Berriman, Zoe Berry, Kimberley Best, Ans-Mari Bester, Yvonne Beuvink, Emily Bevan, Sarah Bevins, Tom Bewick, Andrew Bexley, Sonay Beyatli, Fenella Beynon, Arjun Bhadi, Sanjay Bhagani, Shiv Bhakta, Rekha Bhalla, Khushpreet Bhandal, Kulbinder Bhandal, Ashwin Bhandari, Sangam Bhandari, Aashutosh Bhanot, Ravina Bhanot, Prashanth Bhat, Nikhil Bhatia, Rahul Bhatnagar, Karan Bhatt, Janki Bhayani, Deepika Bhojwani, Salimuzzaman Bhuiyan, Anna Bibby, Fatima Bibi, Naheeda Bibi, Salma Bibi, Tihana Bicanic, Sarah Bidgood, Julie Bigg, Sarah Biggs, Alphonsa Biju, Andras Bikov, Sophie Billingham, Jessica Billings, Martin Binney, Alice Binns, Muhammad BinRofaie, Oliver Bintcliffe, Catherine Birch, Jenny Birch, Katherine Birchall, Sam Bird, Sumedha Bird, Mark Birt, Kilanalei Bishop, Linda Bishop, Lisa Bishop, Karen Bisnauthsing, Nibedan Biswas, Sahar Biuk, Karen Blachford, Ethel Black, Helen Black, Karen Black, Mairead Black, Polly Black, Hayley Blackgrove, Bethan Blackledge, Joanne Blackler, Samantha Blackley, Helen Blackman, Caroline Blackstock, Loraine Blackwood, Francesca Blakemore, Helen Blamey, Alison Bland, Sujata Blane, Simon Blankley, Parry Blaxill, Katie Blaylock, Jane Blazeby, Natalie Blencowe, Ben Bloom, Jack Bloomfield, Angela Bloss, Hannah Bloxham, Louise Blundell, Andrew Blunsum, Mark Blunt, Ian Blyth, Kevin Blyth, Andrew Blythe, Karen Blythe, Marilyn Boampoaa, Boniface Bobie, Karen Bobruk, Pritesh Bodalia, Neena Bodasing, Tanya Bodenham, Gabriele Boehmer, Marta Boffito, Kristyna Bohmova, Sumit Bokhandi, Maria Bokhar, Saba Bokhari, Sakina Bokhari, Syed O Bokhari, Ambrose Boles, Charlotte Bond, Helena Bond, Stuart Bond, Thomas Bond, Alice Bone, Georgia Boniface, Lizzy Bonney, Joanne Borbone, Naomi Borman, Fiona Bottrill, Laura Bough, Hayley Boughton, Zoe Boult, Miriam Bourke, Stephen Bourke, Michelle Bourne, Rachel Bousfield, Lucy Boustred, Alexandra Bowes, Amy Bowes, Philip Bowker, Louise Bowman, Simon Bowman, Rachel Bowmer, Angie Bowring, Helen Bowyer, Jenny Boyd, Laura Boyd, Namoi Boyle, Pauline Boyle, Rosalind Boyle, Louise Boyles, Leanna Brace, Jodie Bradder, Clare Jane Bradley, Pamela Bradley, Patrick Bradley, Paul Bradley, Joanne Bradley-Potts, Lynne Bradshaw, Zena Bradshaw, Rebecca Brady, Shirin Brady, Denise Braganza, Marie Branch, Thomas Brankin-Frisby, Jamie Brannigan, Louise Brassington, Sophie Brattan, Fiona Bray, Nancy Bray, Manny Brazil, Lucy Brear, Tracy Brear, Stephen Brearey, Laura Bremner, Morwenna Brend, Giovanna Bretland, Chris Brewer, Hannah Bridge, Gavin Bridgwood, Sara Brigham, John Bright, Christopher E Brightling, Lutece Brimfield, Elaine Brinkworth, Robin Brittain-Long, Vianne Britten, Lauren Broad, Sarah Broad, Rosie Broadhurst, Andrew Broadley, Marie Broadway, Christopher Brockelsby, Megan Brocken, Tomos Brockley, Mary Brodsky, Fiona Brogan, Liz Brohan, Felicity Brokke, Jacob Brolly, David Bromley, Hannah Brooke-Ball, Verity Brooker, Matthew Brookes, Alison Brooks, Karen Brooks, Nicole Brooks, Philip Brooks, Rachel Brooks, Sophie Brooks, Natalie Broomhead, Chloe Broughton, Nathaniel Broughton, Matt Brouns, Alison Brown, Ammani Brown, Carly Brown, Catrin Brown, Ellen Brown, Heather Brown, Janet Brown, Louise Brown, Niall Brown, Pauline Brown, Richard Brown, Robert Brown, Steven Brown, Tom Brown, Bria Browne, Charlotte Browne, Duncan Browne, Mitchell Browne, Stephen Brownlee, Alba Brraka, David Bruce, Johanna Bruce, Michelle Bruce, Wojciech Brudlo, Nigel Brunskill, Alan Brunton, Margaret Brunton, Mandy Bryan, Meera Bryant, April Buazon, Maya H Buch, Ruaridh Buchanan, Danielle Buche, Amanda Buck, Matthew Buckland, Laura Buckley, Philip Buckley, Sarah Buckley, Carol Buckman, Kathleen Buckmire, George Bugg, Ramadan Bujazia, Marwan Bukhari, Shanze Bukhari, Richard Bulbulia, Alex Bull, Damian Bull, Rhian Bull, Thomas Bull, Naomi Bulteel, Kasun Bumunarachchi, Roneleeh Bungue-Tuble, Caroline Burchett, Dorota Burda, Christy Burden, Thomas G Burden, Mika Burgess, Richard Burgess, Sophia Burgess, Paula Burgett, Adrian Burman, Sara Burnard, Caroline Burnett, Amanda Burns, Amy Burns, Collette Burns, James Burns, Karen Burns, Daniel Burrage, Kate Burrows, Claire Burston, Ben Burton, Fiona Burton, Matthew Burton, Deborah Butcher, Aaron Butler, Jessica Butler, Joanne Butler, Joshua Butler, Peter Butler, Susan Butler, Al-Tahoor Butt, Mohammad M Butt, Caryl Butterworth, Nicola Butterworth-Cowin, Robert Buttery, Tom Buttle, Heather Button, Daniel Buttress, Jane Byrne, Wendy Byrne, Victoria Byrne-Watts, Eleanor Byworth, Amanda Cabandugama, Ruth Cade, Anthony Cadwgan, Donna Cairney, James Calderwood, Darren Caldow, Giorgio Calisti, Debbie Callaghan, Jennifer Callaghan, Claire Callens, Donaldson Callum, Caroline Calver, Melissa Cambell-Kelly, Tracey Camburn, David R Cameron, Eleanor Cameron, Fraser Cameron, Sheena Cameron, Christian Camm, Renee F D Cammack, Alison Campbell, Amy Campbell, Barbara Campbell, Bridget Campbell, Debbie Campbell, Helen Campbell, Hilary Campbell, Jonathan Campbell, Mark Campbell, Robyn Campbell, Wynny Campbell, Quentin Campbell Hewson, Julie Camsooksai, Lisa Canclini, Shaula M Candido, Janie Candlish, Cielito Caneja, Johnathon Cann, Ruby Cannan, Emma Cannon, Michael Cannon, Petra Cannon, Vivienne Cannons, Jane Cantliff, Ben Caplin, Santino Capocci, Noemi Caponi, Angelika Capp, Anne Capps-Jenner, Thomas Capstick, Ishmael Carboo, Mary Cardwell, Rachel Carey, Simon Carley, Tammy Carlin, Samantha Carmichael, Mandy Carnahan, Charlotte Caroline, Jodi Carpenter, Sharon Carr, Anna Carrasco, Zoe Carrington, Paul Carroll, Anne Carstairs, Jonathan Carter, Michael Carter, Paul Carter, Penny Carter, Steven Carter, Douglas Cartwright, Jo-Anne Cartwright, Claire Carty, Sinead Carty, Jaime Carungcong, Susan Casey, Annie Cassells, Barbara Cassimon, Teresa Castiello, Gail Castle, Bridget Castles, Melanie Caswell, Ana Maria Catana, Heidi Cate, Susanne Cathcart, Katrina Cathie, Christine Catley, Laura Catlow, Matthew Caudwell, Jill Caulfield, Anna Cavazza, Luke Cave, Simon Cavinato, Frianne Cawa, Kathryn Cawley, Chloe Caws, Hankins Cendl, Hannah Century, Jeva Cernova, Mansur Cesay, Ed Cetti, Stephanie Chabane, Manish Chablani, Cathleen Chabo, David Chadwick, Julie Chadwick, Robert Chadwick, Ela Chakkarapani, Arup Chakraborty, Mallinath Chakraborty, Mollika Chakravorty, James Chalmers, Richard Chalmers, Georgina Chamberlain, Sarah Chamberlain, Emma Chambers, Jonathan Chambers, Lucy Chambers, Naomi Chambers, Alex Chan, Carmen Chan, Cheuk Chan, Evelyn Chan, Kayen Chan, Kimberley Chan, Ping Chan, Rebekah (Pui-Ching) Chan, Xin Hui Chan, Chris Chandler, Heidi Chandler, Kim Jessica Chandler, Stuart Chandler, Zoe Chandler, Sumit Chandra, Navin Chandran, Badrinathan Chandrasekaran, Yvonne Chang, Josephine Chaplin, Graeme Chapman, John Chapman, Katie Chapman, Laura Chapman, Lianne Chapman, Polly Chapman, Timothy Chapman, Lucy Chappell, Linda Chapple, Amanda Charalambou, Bethan Charles, Dianne Charlton, Kevin Chatar, Calvin Chatha, Ritesh Chaube, Muhammad Y N Chaudhary, Iram Chaudhry, Nazia Chaudhuri, Muhammad Chaudhury, Anoop Chauhan, Ruchi S Chauhan, Vipul Chauhan, Nicola Chavasse, Vipal Chawla, Lindsay Cheater, James Cheaveau, Charlotte Cheeld, Michelle Cheeseman, Fang Chen, Hui M Chen, Terence Chen, Lok Y Cheng, Zhihang Cheng, Helen Chenoweth, Chun H Cheong, Shiney Cherian, Mary Cherrie, Helen Cheshire, Barry Chesterson, Chee K Cheung, Elaine Cheung, Michelle Cheung, Claire Cheyne, Swati Chhabra, Wei L Chia, Eric Chiang, Angela Chiapparino, Rosavic Chicano, Zviedzo A Chikwanha, Sam Chilcott, Phillipa Chimbo, KokWai Chin, Wen J Chin, Rumbidzai Chineka, Amol Chingale, Vashira Chiroma, Heather Chisem, Claire Chisenga, Ben Chisnall, Carolyn Chiswick, Sunder Chita, Nihil Chitalia, Matthew Chiu, Brenda Chivima, Catherine Chmiel, Soha Choi, Willy Choon Kon Yune, Vandana Choudhary, Sarah Choudhury, Bing-Lun Chow, Mahibbur Chowdhury, Shahid Chowdhury, Victoria Christenssen, Peter Christian, Alexander Christides, Fiona Christie, Daniel Christmas, Thereza Christopherson, Mark Christy, Paris Chrysostomou, Yunli Chua, Dip Chudgar, Richard Chudleigh, Srikanth Chukkambotla, Michael Eze Chukwu, Izu Chukwulobelu, Chi Y Chung, Elaine Church, Sara R Church, David Churchill, Nicole Cianci, Paola Cicconi, Paola Cinardo, Zdenka Cipinova, Bessie Cipriano, Sarah Clamp, Melanie Clapham, Edel Clare, Sarbjit Clare, Andrew Clark, Charlotte Clark, Diane Clark, Felicity Clark, Gabrielle Clark, James Clark, Katherine Clark, Kaylea Clark, Louise Clark, Lucy Clark, Matthew Clark, Patricia Clark, Richard Clark, Thomas Clark, Zoe Clark, Andrea Clarke, Heather Clarke, Paul Clarke, Robert Clarke, Roseanne Clarke, Samantha Clarke, Sheron Clarke, Tracy Clarke, Alleyna Claxton, Kate Clay, Elizabeth Clayton, Olivia Clayton, Jill Clayton-Smith, Chris Cleaver, Carlota Clemente de la Torre, Jayne Clements, Suzanne Clements, Rachael Clifford, Sarah Clifford, Amelia Clive, Samantha Clueit, Andrea Clyne, Michelle Coakley, Peter G L Coakley, Tony Coates, Kathryn Cobain, Alexandra Cochrane, Patricia Cochrane, Maeve Cockerell, Helen Cockerill, Shirley Cocks, Rachel Codling, Adam Coe, Samantha Coetzee, David Coey, Danielle Cohen, Jonathan Cohen, Oliver Cohen, Mike Cohn, Louise Coke, Olutoyin Coker, Nicholas Colbeck, Roghan Colbert, Esther Cole, Jade Cole, Joby Cole, Julie Cole, Richard Cole, Garry Coleman, Matt Coleman, Holly Coles, Macleod Colin, Alicia Colino-Acevedo, Julie Colley, Dawn Collier, Heather Collier, Paul Collini, Emma Collins, Jaimie Collins, Joanne Collins, Nicola Collins, Sally Collins, Vicky Collins, Andrew Collinson, Bernadette Collinson, Jennifer Collinson, Matthew Collis, Madeleine Colmar, Hayley Emma Colton, James Colton, Katie Colville, Carolyn Colvin, Ryan Colwell, Edward Combes, David Comer, Alison Comerford, Dónal Concannon, Robin Condliffe, Lynne Connell, Natalie Connell, Karen Connelly, Gavin Connolly, Emma Connor, Antonia Conroy, Veronica Conteh, Rory Convery, Francesca Conway, Grainne Conway, Rhiannon Conway, Jo-Anna Conyngham, Colette Cook, Eloise Cook, Gemma Cook, Helen Cook, Julie Cook, Danielle Cooke, Graham Cooke, Katrina Cooke, Tim Cooke, Adele Cooper, Chris Cooper, David Cooper, Helen Cooper, Jamie Cooper, Joanne Cooper, Joshua Cooper, Lauren Cooper, Nick Cooper, Rowena Cooper, Thomas Cope, Sinead Corbet, Carolyn Corbett, John Corcoran, Chris Cordell, Jessica Cordle, Alasdair Corfield, John Corless, Alison Corlett, Joe Cornwell, Michael Cornwell, Diana Corogeanu, Mirella Corredera, Ruth Corrigan, Rita Corser, Denise Cosgrove, Tracey Cosier, Patricia Costa, Charlie Coston, Susannah Cotgrove, Zoe Coton, Lisa-Jayne Cottam, Rhiannon Cotter, Donna Cotterill, Caroline Cotton, Katy Cotton, Andrew Coull, James Coulson, David Counsell, David Counter, Cherry Coupland, Ellie Courtney, Julia Courtney, Rebecca Cousins, Alexander Cowan, Elena Cowan, Richard Cowell, Louise Cowen, Steve Cowman, Amanda Cowton, Ellie Cox, Giles Cox, Karina Cox, Miriam Cox, Karen Coy, Beverly Craig, Victoria Craig, Felicity Craighead, Matthew Cramp, Jacolene Crause, Adrian Crawford, Angie Crawford, Emma Crawford, Isobel Crawford, Richard Crawforth, Sarah Crawshaw, Ben Creagh-Brown, Andrew Creamer, Joanne Cremona, Saveria Cremona, Janet Cresswell, Mark Cribb, Charles Crichton, Declan Crilly, Lauren Crisp, Nikki Crisp, Dominic Crocombe, Maria Croft, Jennifer Crooks, Harriet Crosby, Tim Cross, Amy Crothers, Stephen Crotty, Susan Crouch, Madeleine Crow, Amanda Crowder, Kate Crowley, Teresa Crowley, Rebecca Croysdill, Callum Cruickshank, Conor Cruickshank, Irena Cruickshank, James Cruise, Carina Cruz, Trino Cruz Cervera, Dominic Cryans, Guanguo Cui, Helen Cui, Lorraine Cullen, Gillian Cummings-Fosong, Marie Cundall, Victoria Cunliffe, Lorraine Cunningham, Neil Cunningham, Nicola Cunningham, Jason Cupitt, Hollie Curgenven, David Curran, Simon Curran, Craig Currie, Jacqueline Currie, Scarlett Currie, Jonathan Curtis, Katrina Curtis, Olivia Curtis, Thomas Curtis, Rebecca Cuthbertson, Sean Cutler, Marta Czekaj, Patrycja Czylok, Dhanishta D Ramdin, Joana da Rocha, Andrew Dagens, Helen Daggett, Jacqui Daglish, Sandeep Dahiya, Anne Dale, Katie Dale, Michaela Dale, Sam Dale, Jolyon Dales, Helen Dalgleish, Helen Dallow, Dermot Dalton, Zoe Daly, Eleanor Damian, Akila Danga, Amelia Daniel, Priya Daniel, Allison Daniels, Adela Dann, Sandra Danso-Bamfo, Nimo Daoud, Alex Darbyshire, Janet Darbyshire, Paul Dargan, Paul Dark, Kate Darlington, Tom Darton, Guledew Darylile, Manjusha Das, Sukamal Das, Martin Daschel, Joanne Dasgin, Dibyendu Datta, Anna Daunt, Emily Davenport, Mark Davey, Miriam Davey, Molly Davey, Mini David, Alexander Davidson, Laura Davidson, Neil Davidson Davidson, Richard Davidson, Albert Davies, Alison Davies, Amanda Davies, Amy Davies, Angela Davies, Carolyn Davies, Catrin Davies, Cheryl Davies, Drew Davies, Elaine Davies, Ffyon Davies, Helen Davies, Jeni Davies, Jim Davies, Karen Davies, Kelly Davies, Kim Davies, Louisa Davies, Matthew Davies, Michelle Davies, Nina Davies, Owen Davies, Patrick Davies, Rachel Davies, Rhys Davies, Ruth Davies, Sarah Davies, Simon Davies, Alison Davis, Gwyneth Davis, Illinos Davis, Julie-Ann Davis, Katherine Davis, Peter Davis, Alexander Davison, Mark Davy, Christine Dawe, H Dawe, Mark Dawkins, Danielle Dawson, Elizabeth Dawson, Joy Dawson, Susan Dawson, Tom Dawson, Andrew Daxter, Andrew Day, Jacob Day, Jeremy Day, Jamie D'Costa, Parijat De, Duneesha de Fonseka, Toni de Freitas, Frederico De Santana Miranda, Eleanor de Sausmarez, Shanika de Silva, Thushan de Silva, Jessica De Sousa, Paulo De Sousa, James de Souza, Anthony de Soyza, Natasha de Vere, Johannes de Vos, Bethan Deacon, Sharon Dealing, Anna Dean, Julie Dean, Katrina Dean, Stephen Dean, Tessa Dean, Jill Deane, James Dear, Effie Dearden, Catherine Deas, Samuel Debbie, Gabor Debreceni, Vashist Deelchand, Matthew Deeley, Joanne Deery, Emmanuel Defever, Manuela Del Forno, Arnold Dela Rosa, Amanda Dell, Carrie Demetriou, David DeMets, Jane Democratis, Jacqueline Denham, Emmanuelle Denis, Laura Denley, Craig Denmade, Kathy Dent, Martin Dent, Elise Denton, Tom Denwood, Nishigandh Deole, Darshita Depala, Maria Depante, Susan Dermody, Amisha Desai, Asmita Desai, Purav Desai, Sanjeev Deshpande, Vai Deshpande, Sirjana Devkota, Prakash Dey, Vishal Dey, Rogin Deylami, Kevin Dhaliwal, Sundip Dhani, Amandeep Dhanoa, Mili Dhar, Devesh Dhasmana, Ekanjali Dhillon, Reiss Dhillon, Priya Dias, Stephanie Diaz, Kayleigh Diaz-Pratt, Debbie Dickerson, Pamela Dicks, Stuart Dickson, Sean Dillane, Sarah Diment, ThaiHa Dinh, Alex Dipper, Laura Dirmantaite, Lisa Ditchfield, Sarah Diver, Lavanya Diwakar, Caroline Dixon, Giles Dixon, Brice Djeugam, Petr Dlouhy, Paul Dmitri, Laurence Dobbie, Marinela Dobranszky Oroian, Charlotte Dobson, Lee Dobson, Marie Docherty, David Dockrell, James Dodd, Jackie Dodds, Rebecca Dodds, Steve Dodds, Richi Dogra, Erin Doherty, Warren Doherty, Yumiko Doi, Iain Doig, Eleanor Doke, Daniel Dolan, Mark Dolman, Rozzie Dolman, Lisa Donald, Callum Donaldson, Christopher Donaldson, Denise Donaldson, Gillian Donaldson, Kate Donaldson, Joanne Donnachie, Christopher Donnelly, Eilish Donnelly, Ronan Donnelly, Aravindhan Donohoe, Gemma Donohoe, Bryan Donohue, Sinead Donton, Emma Dooks, Grainne Doran, Kane Dorey, Sharon Dorgan, Moonira Dosani, Davinder Dosanjh, Paula Dospinescu, Katie Douglas, Jonathan Douse, Lucy Dowden, Michelle Dower, Kerry Dowling, Sud Dowling, Nicola Downer, Charlotte Downes, Rob Downes, Thomas Downes, Damian Downey, Robert Downey, Louise Downs, Simon Dowson, Cornel Dragan, Cristina Dragos, Maire Drain, Chelsea Drake, Victoria Drew, Olivia Drewett, Celine Driscoll, Helena Drogan, Graham Drummond, Ronald Druyeh, Simon Drysdale, An Du Thinh, Hazel Dube, Judith Dube, Stephen Duberley, Hayley Duckles-Leech, Nicola Duff, Emma Duffield, Helen Duffy, Lionel Dufour, Annette Duggan, Parveen Dugh, Janice Duignan, Simon Dummer, Andrew Duncan, Christopher Duncan, Fullerton Duncan, Gregory Duncan, Stephanie Dundas, Alessia Dunn, Charlotte Dunn, Damian Dunn, Laura Dunn, Paul Dunn, Charlene Dunne, Karen Dunne, Fiona Dunning, Aidan Dunphy, Venkat Duraiswamy, Beatriz Duran, Ingrid DuRand, Natalie Duric, Alison Durie, Emily Durie, Hannah Durrington, Haris Duvnjak, Akshay Dwarakanath, Laasya Dwarakanath, Ellen Dwyer, Claudia Dyball, Kristyn Dyer, Harvey Dymond, Tom Dymond, Chris Eades, Laura Eagles, Joanne Early, Melissa Earwaker, Nicholas Easom, Clare East, Amy Easthope, Fraser Easton, Ruth Eatough, Oluwadamilola Ebigbola, Martin Ebon, Sinan Eccles, Chloe Eddings, Michael Eddleston, Maureen Edgar, Katharine Edgerley, Nicholas Edmond, Julie Edmonds, Mary Edmondson, Tracy Edmunds, Alexandra Edwards, Catherine Edwards, Joy Edwards, Kennedy Edwards, Mandy Edwards, Tomos Edwards, Jenny Eedle, Dawn Egginton, Loveth Ehiorobo, Sarah Eisen, Ugochukwu Ekeowa, Mohamed Ekoi, Ayomide Ekunola, Soha El Behery, Mohamed Elbeshy, Kate El-Bouzidi, Jennifer Elder, Mohammed El-Din, Diana Eleanor, Ibrahim Eletu, Eman Elfar, Mayy M Elgamal, Amr Elgohary, Stellios Elia, Jennifer Elias, Tania Elias, Nadia Elkaram, Mohammed El-Karim, Andrew V Elkins, Julie Ellam, Nikki Ellard, Laura N Ellerton, Lucy Elliot, Amy Elliott, Chris Elliott, Fiona Elliott, Kerry Elliott, Scott Elliott, Annie Ellis, Christine Ellis, Kaytie Ellis, Tak-Yan Ellis, Yvette Ellis, Megan Ellison, Rahma Elmahdi, Einas Elmahi, Hannah-May Elmasry, Mohamed Elmi, Najla Elndari, Omer Elneima, Mohamed Elokl, Ahmed Elradi, Mohamed Elsaadany, Sally El-Sayeh, Hana El-Sbahi, Tarek Elsefi, Karim El-Shakankery, Robert Elshaw, Hosni El-Taweel, Sarah Elyoussfi, Jonathan Emberey, Jonathan R Emberson, John Emberton, Julian Emmanuel, Ingrid Emmerson, Michael Emms, Florence Emond, Marieke Emonts, Nicu Enachi, Angila Engden, Katy English, Emma Entwistle, Hene Enyi, Marios Erotocritou, Peter Eskander, Hanif Esmail, Brynach Evans, Chris Evans, Debra evans, Gail Evans, Gareth Evans, Jennifer Evans, Lisa Evans, Lynn Evans, Margaret Evans, Mim Evans, Morgan Evans, Ranoromanana Evans, Teriann Evans, Terry J Evans, Caroline Everden, Serenydd Everden, Hayley Evison, Lynsey Evison, Penny Eyton-Jones, Jacqueline Faccenda, Leila Fahel, Youstina Fahmay, Sara Fairbairn, Terry Fairbairn, Andy Fairclough, Louise Fairlie, Mark Fairweather, Anne Fajardo, Naomi Falcone, Euan Falconer, Jonathan Falconer, John Fallon, Andrea Fallow, David Faluyi, Victoria Fancois, Qayyum Farah, Nowin Fard, Amr Farg, Margaret Farinto, Adam Farmer, Katie Farmer, Toni Farmery, Samantha Farnworth, Faiyaz Farook, Hadia Farooq, Sidrah Farooq, Fiona Farquhar, Karen Farrar, Aaron Farrell, Barbara Farrell, James Farthing, Syeda Farzana, Rahmatu Fasina, Azam Fatemi, Mina Fatemi, Nibah Fatimah, Maria Faulkner, Saul N Faust, Joe Fawke, Sinmidele Fawohunre, Abul Fazal, Simon Fearby, Alex Feben, Federico Fedel, Daria Fedorova, Christopher Fegan, Mae Felongco, Lynsey Felton, Tim Felton, Kate Fenlon, Andrea Fenn, Isabelle Fenner, Ciara Fenton, Melisa Fenton, Cameron Ferguson, Jenny Ferguson, Kathryn Ferguson, Katie Ferguson, Susan Ferguson, Susie Ferguson, Victoria Ferguson, Denzil Fernandes, Candida Fernandez, Eduardo Fernandez, Maria Fernandez, Sonia Fernandez Lopez, Callum Jeevan Fernando, Ahmed Feroz, Pietro Ferranti, Thais Ferrari, Eleanor Ferrelly, Alexandra Ferrera, Emma Ferriman, Nicholas Fethers, Ben Field, Janet Field, Rebecca Field, Karen Fielder, Lindsey Fieldhouse, Andra Fielding, Julie Fielding, Len Fielding, Sarah Fielding, Asma Fikree, Sarah Ann Filson, Sarah Finbow, Debbie Finch, Joanne Finch, Laurie Finch, Natalie Fineman, Lauren Finlayson, Adam Finn, Joanne Finn, Clare Finney, Sofia Fiouni, Jo Fiquet, James Fisher, Neil Fisher, Daniel Fishman, Krystofer Fishwick, Lorraine Fitzgerald, Jan Flaherty, Michael Flanagan, Charles Flanders, Julie Fleming, Lucy Fleming, Paul Fleming, William Flesher, Alison Fletcher, Jonathan Fletcher, Lucy Fletcher, Simon Fletcher, Sophie Fletcher, Karen Flewitt, Christopher Flood, Ian Floodgate, Vincent Florence, Sharon Floyd, Rachel Flynn, Claire Foden, Adama Fofana, Georgina Fogarty, Paul Foley, Linda Folkes, Daniela M Font, Aiwyne Foo, Jane Foo, Andrew Foot, Jayne Foot, Jane Forbes, Jamie Ford, Jennifer Foreman, Caroline Fornolles, Adam Forrest, Ellie Forsey, Miranda Forsey, Thomas Forshall, Elliot Forster, Julian Forton, Emily Foster, Joseph Foster, Rachel Anne Foster, Tracy Foster, Theodora Foukanelli, Angela Foulds, Ian Foulds, Folakemi Fowe, Emily Fowler, Robert Fowler, Stephen Fowler, Caroline Fox, Claire Fox, Heather Fox, Jonathan Fox, Lauren Fox, Natalie Fox, Olivia Fox, Simon Fox, Sarah-Jane Foxton, Rebecca Frake, Alex Francioni, Olesya Francis, Rebecca Francis, Sarah Francis, Theodora Francis-Bacon, Jason Frankcam, Helen Frankland, Jessica Franklin, Catherine Fraser, Sharon Frayling, Martyn Fredlund, Matthew Free, Carol Freeman, Elaine Freeman, Hannah Freeman, Nicola Freeman, Clare Freer, Eleanor French, Matthew Frise, Renate Fromson, Claire Froneman, Adam Frosh, John Frost, Victoria Frost, Oliver Froud, Rachel Frowd, Arun Fryatt, Janet Fu, Bridget Fuller, Liz Fuller, Neil Fuller, Tracy Fuller, Duncan Fullerton, Jenny Fullthorpe, Carrie Fung, Gayle Fung, Sarah Funnell, John Furness, Andrew Fyfe, Nytianandan G, Elizabeth Gabbitas, Claire Gabriel, Diana Gabriel, Hadiza Gachi, Joshua Gahir, Sarveen Gajebasia, Katarzyna Gajewska-Knapik, Christopher Gale, Hugo Gale, Rebecca Gale, Swetha Gali, Bernadette Gallagher, Jude Gallagher, Rosie Gallagher, William Gallagher, Joanne Galliford, Catherine Galloway, Chris Galloway, Emma Galloway, Jacqui Galloway, James Galloway, Laura Gamble, Liz Gamble, Brian Gammon, Jaikumar Ganapathi, Ramesh Ganapathy, Kaminiben Gandhi, Sarah Gandhi, Usha Ganesh, Abrar Gani, Emma-James Garden, Antoni Dariusz Gardener, Emma Gardiner, Michael Gardiner, Phil Gardiner, Siobhan Gardiner, Caroline Gardiner-Hill, Jonathan Gardner, Mark Garfield, Atul Garg, Nathan Garlick, Justin Garner, Lucie Garner, Zoe Garner, Kim Garnett, Rosaline Garr, Florence Garty, Rachel Gascoyne, Hyeriju Gashau, Aoife Gatenby, Erin Gaughan, Alok Gaurav, Mariana Gavrila, Jane Gaylard, Emma Gaywood, Catherine Geddie, Ian Gedge, Sarah Gee, Minerva Gellamucho, Karzan Gelly, Leila Gelmon, Sandra Gelves-Zapata, Gemma Genato, Susan Gent, Natalie Geoghegan, Sam George, Tina George, Simon Georges, Domonique Georgiou, Peter Gerard, Leigh Gerdes, Louise Germain, Helen Gerrish, Abel Getachew, Louise Gethin, Hisham Ghanayem, Amardeep Ghattaoraya, Anca Gherman, Alison Ghosh, Justin Ghosh, Sudhamay Ghosh, Sarra Giannopoulou, Malick Gibani, Ben Gibbison, Kerry Gibbons, Alex Gibson, Bethan Gibson, Kimberley Gibson, Kirsty Gibson, Sian Gibson, Cat Gilbert, Jeanette Gilbert, Joanne Gilbert, Kayleigh Gilbert, Benjamin Giles, Mandy Gill, Lynne Gill, Paul Gillen, Annelies Gillesen, Katherine Gillespie, Elizabeth Gillham, Andrew Gillian, Deborah Gilliland, Robert Gillott, Danielle Gilmour, Kate Gilmour, Theodora Giokanini-Royal, Anna Gipson, Joanna Girling, Rhian Gisby, Angelena Gkioni, Aikaterini Gkoritsa, Effrossyni Gkrania-Klotsas, Amy Gladwell, James Glanville, Jessica Glasgow, Susannah Glasgow, Jon Glass, Lynn Glass, Sharon Glaysher, Lisa Gledhill, Ana Glennon, John Glover, Kyle Glover, Jan Glover Bengtsson, Chevanthy Gnanalingam, Julie Goddard, Wendy Goddard, Emily Godden, Jo Godden, Emma Godson, Sukanya Gogoi, Aiky Goh, Rebeca Goiriz, Sriya Gokaraju, Raphael Goldacre, Arthur Goldsmith, Portia Goldsmith, Darren Gomersall, Lucia Gomez, Raquel Gomez-Marcos, Ali Gondal, Celia Gonzalez, Jack Goodall, Bob Goodenough, Laura Goodfellow, James Goodlife, Camelia Goodwin, Elizabeth Goodwin, Jayne Goodwin, Paula Goodyear, Rajiv Gooentilleke, Michelle Goonasekara, Sheila Gooseman, Shameer Gopal, Sally Gordon, Hugh Gorick, Caitlin Gorman, Claire Gorman, Stuart Gormely, Diana Gorog, Michelle Gorst, Thomas Gorsuch, Jayshreebahen Gosai, Rebecca Gosling, Sally Gosling, Georgina Gosney, Vanessa Goss, Dzintars Gotham, Naomi Gott, Elizabeth Goudie, Angela Gould, Susan Gould, Lysander Gourbault, Abha Govind, Sharon Gowans, Girish Gowda, Rohit Gowda, Hannah Gower, Thomas Gower, Pankaj Goyal, Sunil Goyal, Sushant Goyal, Clive Graham, Jonathan Graham, Justin Graham, Libby Graham, Sharon Graham, Matthew Graham-Brown, Julia Grahamslaw, Gianluca Grana, Tracyanne Grandison, Louis Grandjean, Alison Grant, Ann Grant, David Grant, Matthew Grant, Pauleen Grant, Rhys Gravell, Jenny Graves, Alasdair Gray, Catherine Gray, Georgina Gray, Jackie Gray, Karen Gray, Nicola Gray, Sebastian Gray, Alan Grayson, Fiona Greaves, Paul Greaves, Charlotte Green, Christopher Green, David Green, Frederick Green, Joel Green, Marie Green, Nicola Green, Stacey Green, Teresa Green, Diarra Greene, Philippa Greenfield, Alan Greenhalgh, Daniel Greenwood, Sandra Greer, James Gregory, Jane Gregory, Katie Gregory, Tamsin Gregory, Jill Greig, Julia Greig, Rebecca Grenfell, Teena Grenier, Susan Grevatt, Glaxy Grey, Andrew Gribbin, Amy Gribble, Natasha Grieg, Douglas Grieve, Ben Griffin, Denise Griffin, Mel Griffin, Sian Griffith, Andrew Griffiths, Daniel Griffiths, David Griffiths, Donna Griffiths, Isabel Griffiths, Mark Griffiths, Nicola Griffiths, Oliver Griffiths, Sarah Griffiths, Yvonne Griffiths, Sofia Grigoriadou, Steph Grigsby, Evelina Grobovaite, Rachel Groome, Liliana Grosu, Jenny Grounds, Margaret Grout, Helen Grover, Jayne Groves, Neil Grubb, Julie Grundy, Francesca Guarino, Sharada Gudur, Sharazeq Guettari, Shivang Gulati, Vikas Gulia, Pumali Gunasekera, Malin Gunawardena, Kirun Gunganah, Jessica Gunn, Emma Gunter, Alok Gupta, Atul Gupta, Rajeev Gupta, Richa Gupta, Rishi Gupta, Tarun Gupta, Vineet Gupta, Ankur Gupta-Wright, Victoria Guratsky, Alvyda Gureviciute, Sambasivarao Gurram, Bhawana Gurung, Shraddha Gurung, Hazel Guth, Ruth Habibi, Berkin Hack, Pamela Hackney, Christian Hacon, Aiman Haddad, Denise Hadfield, Michalis Hadjiandreou, Nikolaos Hadjisavvas, Anna Haestier, Nauman Hafiz, Rana Hafiz-Ur-Rehman, Javed Hafsa, Samantha Hagan, Jack William Hague, Rosemary Hague, Kate Haigh, Christina Haines, Scott Hainey, Morton Hair, Brigid Hairsine, Juraj Hajnik, Anne Haldeos, Writaja Halder, Jennie Hale, Carmel Halevy, Paul Halford, William Halford, Alistair Hall, Anthony Hall, Claire Hall, Elizabeth Hall, Fiona Hall, Helen Hall, Jennifer Hall, Kathryn Hall, Jan Hallas, Kyle Hallas, Charles Hallett, Becky-Lee Halls, Heather Halls, Maryam Hamdollah-Zadeh, Bilal Hameed, Imran Hamid, Mohamad Hamie, Bethany Hamilton, Fergus Hamilton, Leigh Hamilton, Nicola Hamilton, Ruth Hamlin, Eleanor Hamlyn, Beatrice Hammans, Shirley Hammersley, Kate Hammerton, Bev Hammond, Leah Hammond, Fiona Hammonds, Ibrahim Hamoodi, Karen Hampshire, Jude Hampson, Lucy Hampson, Ozan Hanci, Ian Hancock, Sadiyah Hand, Jasmine Handford, Soran Handrean, Sarah Haney, Sheharyar Hanif, E Hanison, Jennifer Hannah, Amy Hannington, Merhej Hannun, Aidan Hanrath, Anita Hanson, Jane Hanson, Kathryn Hanson, Steve Hanson, Mazhar Ul Haq, Ala Haqiqi, Monjurul Haque, Lesley Harden, Zoe Harding, Simon Hardman, Joanna Hardy, Kumar Haresh, Rachel Harford, Beverley Hargadon, Carolyn Hargreaves, James Hargreaves, Alice Harin, Mohammed Haris, Edward Harlock, Paula Harman, Tracy Harman, Mark Harmer, Muhammad A Haroon, Charlie Harper, Heather Harper, Peter Harper, Rosemary Harper, Sarah Harrhy, Sian Harrington, Yasmin Harrington-Davies, Jade Harris, Jess Harris, John Harris, Laura Harris, Marie-Clare Harris, Nichola Harris, Sophie Harris (CTP), David Harrison, Julie Harrison, Laura Harrison, Melanie Harrison, Rowan Harrison, Susie Harrison, Thomas Harrison, Wendy Harrison, Elizabeth Harrod, Ciaran Hart, Dominic Hart, Lisa Hartley, Rosemary Hartley, Ruth Hartley, Tom Hartley, William Hartrey, Phillipa Hartridge, Stuart Hartshorn, Alice Harvey, Angela Harvey, Max Harvey, Catherine Harwood, Helen Harwood, Brigitte Haselden, Kazi Hashem, Mohammed Hashimm, Tadaaki Hashimoto, Imranullah Hashmi, Zena Haslam, Adil Hassan, Ali Hassan, Wagae UI Hassan, Sapna Hassasing, Jane Hassell, Philip Hassell, Alex Hastings, Bethany Hastings, Janice Hastings, Jonathan Hatton, May Havinden-Williams, Stefan Havlik, Daniel B Hawcutt, Kadean Hawes, Liz Hawes, Nicola Hawes, Annie Hawkins, Nancy Hawkins, Tanya Hawkins, Dan Hawley, Ed Hawley-Jones, Edward Haworth, Cathy Hay, Amna Hayat, Jamal Hayat, Mohamed-Riyal Hayathu, Anne Hayes, Jonas Hayes, Kate Hayes, Melony Hayes, Fiona Hayes, Patrick Hayle, Chloe Haylett, Antara Hayman, Melanie Hayman, Matthew Haynes, Richard Haynes, Rachel Hayre, Sarah Haysom, James Hayward, Patrick Haywood, Tracy Hazelton, Phoebe Hazenberg, Zhengmai He, Elizabeth Headon, Carrie Heal, Brendan Healy, Amy Hearn, Angela Heath, Rowan Heath, Diane Heaton, Kerry Hebbron, Gemma Hector, Andy Hedges, Katrine Hedges, Cheryl Heeley, Elaine Heeney, Rajdeep Heire, Ulla Hemmila, Cassie Hemmings, Scott Hemphill, Deborah Hemsley, Abigail Henderson, Jennifer Henderson, Steven Henderson, Natalie Hennesy, Carol Ann Henry, Joanne Henry, Karol Henry, Lavinia Henry, Margo Henry, Natalie Henry, David Henshall, Mike Herbert, Gillian Herdman, Rosaleen Herdman-Grant, Morag Herkes, Emma Heron, William Herrington, Emilia Heselden, Peta Heslop, Simon Hester, Emily Hetherington, Joseph Hetherington, Chamila Hettiarachchi, Pramodh Hettiarachchi, Hayley Hewer, John Hewertson, Anna Hewetson, Sue Hewins, Claire Hewitt, Davina Hewitt, Richard Hewitt, Robert Heyderman, Mathis Heydtmann, Joseph Heys, Jonathan Heywood, Meg Hibbert, John Hickey, Naomi Hickey, Peter Hickey, Alex Hicks, Jenny Hicks, Scott Rory Hicks, Daniel Higbee, Lucy Higgins, Andrew Higham, Martin Highcock, Judith Highgate, Mondy Hikmat, Amanda Hill, Helen Hill, Joanne Hill, Lisa Hill, Phoebe Hill, Uta Hill, Annette Hilldrith, Catherine Hillman-Cooper, Zoe Hilton, Sarah Hinch, Andrew Hindle, Alice Hindmarsh, Paul Hine, Kim Hinshaw, Clare Hird, Jemma Hives, Benson Ho, Michaela Hoare, David Hobden, Gill Hobden, Maria Hobrok, Simon Hobson, Simon Hodge, Lesley Hodgen, Holly Hodgkins, Louise Hodgkinson, Sally Hodgkinson, David Hodgson, Helen Hodgson, Luke Hodgson, Sheila Hodgson, Gemma Hodkinson, Kenneth Hodson, Matthew Hogben, Lucy Hogg, Lee Hoggett, Abigail Holborow, Catherine Holbrook, Melinda Holden, Thomas Holder, Niels Holdhof, Hannah Holdsworth, Lisa Holland, Maureen Holland, Nicky Holland, Marie Hollands, Elizabeth Holliday, Nina Holling, Laszlo Hollos, Simon Holloway, Marcus Hollyer, Amy Holman, Ann Holmes, Benjamin Holmes, Megan Holmes, Raphael Holmes, Rebecca Holmes, Kelly Holroyd, Caroline Holt, Lyndsey Holt, Siobhan Holt, Susie Holt, Alexandra Holyome, Marie Home, Toni Home, Renate Homewood, Kate Hong, Clare Hooper, Samantha Hope, Susan Hope, Bridget Hopkins, Peter W Horby, Stephanie Horler, Anil Hormis, Daniel Hornan, Nicola Hornby, Zoey Horne, Latoya Horsford, Megan Horsford, Mark Horsford, Valana Horsham, Alexander Horsley, Ashley Horsley, Elizabeth Horsley, Sarah Horton, Jane Hosea, Toby Hoskins, Muhammad S Hossain, Rashed Hossain, Maxine Hough, Sarah Hough, Catherine Houghton, Kathryn Houghton, Rebecca Houlihan, Hamish Houston, Tawe Hove, Roseanna Hovvels, Lee How, Laura Howaniec, Laura Howard, Linda Howard, Lucy Howard, Sarah Howard, Stuart Howard, Richard Howard-Griffin, Serena Howe, Mark Howells, Lyn Howie, Kerry Howlett, Josh Hrycaiczuk, Naing Z Htoon, Su Htwe, Ying Hu, Chiang O H Huah, Abby Huckle, Shahzya Huda, Alison Hudak, Lisa Hudig, Heather Hudson, Oli Hudson, Alison Hufton, Alistair Hughes, Emma Hughes, Gareth Hughes, Heather Hughes, Luke Hughes, Rachel Hughes, Rebecca Hughes, Samantha Hughes, Stephen Hughes, Vikki Hughes, Wesley Hughes, Lukas Huhn, Ching Hui, Ruth Hulbert, Diana Hull, Grace Hull, Robert Hull, Amanda Hulme, Peter Hulme, Wendy Hulse, George Hulston, Ryan Hum, Megan Hume, Charlotte Humphrey, Ismay Humphreys, Alasdair Humphries, Joanne Humphries, Fiona Hunt, Kristen Hunt, Luke Hunt, Sophie Hunt, Alexandra Hunter, Karl Hunter, Neil Hunter, George Huntington, Elizabeth Hurditch, Cian Hurley, Katrina Hurley, Mohammed A Husain, Syeda Y Husaini, Coralie Huson, Afreen Hussain, Ibraar Hussain, Ifza Hussain, Mohammad Hussain, Muhammad Hussain, Reda Hussain, Sajid Hussain, Samia Hussain, Sanniah Hussain, Yasmin Hussain, Mohammed Hussam El-Din, Rebecca Hussey, Camille Hutchinson, Dorothy Hutchinson, Elizabeth Hutchinson, John Hutchinson, Claire Hutsby, Paula Hutton, Daniella Hydes, Jamie Hyde-Wyatt, Niamh Hynes, Megan Hyslop, Mazen Ibraheim, Abdalla Ibrahim, Ahmed Ibrahim, Asil Ibrahim, Mohamed Ibrahim, Wadah Ibrahim, Adetokunbo I Idowu, Muhammad Idrees, Hina Iftikhar, Mawara Iftikhar, Chukwuemeka Igwe, Mohammad Ijaz, Amaju Ikomi, Clare Iles, Stamatina Iliodromiti, Mary Ilsley, Lorna Ilves, La'ali Imam-Gutierrez, Christopher Imray, Haider Imtiaz, Jack Ingham, Julie Ingham, Rory Ingham, Tejas Ingle, Jennifer Inglis, Anne Ingram, Luke Ingram, Peter Inns, Ken Inweregbu, Andreea A Ionescu, Ana Ionita, Ilian P Iordanov, Anil Ipe, Madiha Iqbal, Mohammed Iqbal, Faisal Iqbal Sait, Jane Ireland, Robert Irons, Mohannad Irshad, Muhammad S Irshad, Janice Irvine, Val Irvine, Robert Irving, Mina Ishak, Erica IsherwoodOC User, Aminul Islam, Abdurrahman Islim, Ali Ismail, Omar Ismail, Caroline Ison, M'hamedi Israa, Sharon Isralls, Monica Ivan, Chineze Ivenso, Ashleigh Ivy, Sophie Iwanikiw, Karen Ixer, Menaka Iyer, Mia Iyer, Calum Jack, Amanda Jackson, Ben Jackson, Beth Jackson, Ella Jackson, Hayley Jackson, Helen Jackson, Jane Jackson, Lauren Jackson, Melanie Jackson, Nicola Jackson, Shane Jackson, Patricia Jacob, Reni Jacob, Nicola Jacques, Anisa Jafar, Daniel Jafferji, Ali Jaffery, Chandrashekar Jagadish, Vijay Jagannathan, Mandeep Jagpal, Fernandez R Jaime, Neemisha Jain, Seema Jain, Susan Jain, Sanjay Jaiswal, Danyal Jajbhay, Thomas Jaki, Bintou Jallow, Yusuf Jaly, Sabine Jamal, Zeba Jamal, Yasmin Jameel, Albie James, Christie James, Kate James, Lee James, Linda James, Mark James, Nicholas James, Olivia James, Rebecca James, Ruth James, Tracy James, Jack Jameson, Aaron Jamison, Phoebe Jane, Azara Janmohamed, Sabrina Jansz, Deepa Japp, Victor Jardim, Catherine Jardine, Emma Jarnell, Ellie Jarvie, Claire Jarvis, Rosina Jarvis, Patrycja Jastrzebska, Hafsa Javed, Mays Jawad, Lona Jawaheer, Anu Jayachandran, D Jayachandran, Angelina Jayakumar, Deepak Jayaram, Ravi Jayaram, Geeshath Jayasekera, Thilina Jayatilleke, Abi Jayebalan, Saman Jeddi, Vandana Jeebun, Mohammad S Jeelani, Katie Jeffery, Helen Jeffrey, Jenni Jeffrey, Nathan Jeffreys, Benjamin Jeffs, Debbie Jegede, Taylor Jemima, Ifan Jenkin, Alison Jenkins, Christopher Jenkins, David Jenkins, Elinor Jenkins, Sarah Jenkins, Sian Jenkins, Stephen Jenkins, Jacqui Jennings, Louise Jennings, Virginia Jennings, Ellen Jerome, Douglas Jerry, Ellen Jessup-Dunton, Jorge Antonio Jesus Silva, Champa Jetha, Kishan Jethwa, Jeby Jeyachandran, Shaman Jhanji, Khoo Jian, Zhixin Jiao, Laura Jimenez, Ana Jimenez Gil, Jithin Jith, Teishel Joefield, Navraj Johal, Karine Johannessen, Aisyah Johari, Annie John, Anu John, Navin John, Emma Johns, Margaret Johns, Antoinette Johnson, David Johnson, Emma Johnson, Gillian Johnson, Kathryn Johnson, Katie Johnson, Luke Johnson, Mark Johnson, Nelson Johnson, Oliver Johnson, Tracy Johnson, Claire Johnston, Janet Johnston, Laura Johnston, Susan Johnston, Victoria Johnston, Dawn Johnstone, Ed Johnstone, Janet Johnstone, Manohar Joishy, Adam Jones, Alistair Jones, Annabel Jones, Ben Jones, Bryony Jones, Carys Jones, Ceri Jones, Charlotte Jones, Christine E Jones, Debra Jones, Emily Jones, Gareth Jones, Geraldine Jones, Jac Jones, James Jones, Jessica Jones, Jonathon Jones, Julie Jones, Kate E Jones, Laura Jones, Laura M Jones, Louise Jones, Mathew Jones, Nicola Jones, Paul Jones, R E Jones, Rhianna Jones, Samantha Jones, Sophie Jones, Stefanie Jones, Steve Jones, Taya Jones, Tim Jones, Tracey Jones, Ramya Jonnalagadda, Rebecca Jordache, Sanal Jose, Anna Joseph, Rosane Joseph, Sibet Joseph, Dhaara Joshi, Mehul Joshi, Pratichi Joshi, Benz Josiah, Tiffany Joyce, Adriel Ju Wen Kwek, Edward Jude, Parminder Judge, Jessica Juhl, Sirisha Jujjavarapu, Mark Juniper, Edmund Juszczak, Deepthi Jyothish, Kasamu Kabiru Dawa, Mark Kacar, Nikhil Kadam, Rebecca Kahari, Gail Kakoullis, Azad Kala Bhushan, Richard J K Kalayi, Roobala Kaliannan Periyasami, Efthymia Kallistrou, Seika Kalsoom, Elisa Kam, John Kamara, Mohamed Kamara, Ajay Kamath, Prakash Kamath, Ravindra Kamath, Siddharth A Kamerkar, Nick Kametas, Musaiwale Kamfose, Leia Kane, Osei Kankam, Thogulava Kannan, Abhinav Kant, Vikas Kapil, Ritoo Kapoor, Sonal Kapoor, Sourjya Kar, Janaka Kara, Rona Kark, Nicholas Karunaratne, Natashja Kasianczuk, Vidya Kasipandian, Rizwan Kassam, Janarth Kathirgamachelvam, Victoria Katsande, Kulbinder Kaul, Daljit Kaur, Dervinder Kaur, Jasmin Kaur, Jaspreet Kaur, Zunaira Kausar, Mohammad A Kawser, Andrea Kay, Sarah Kay, Jossy N Kayappurathu, Callum Kaye, Ahemd Kazeem, Naved Kazi, Rachel Kearns, Nichola Kearsley, Joanne Keating, John Keating, Liza Keating, Elizabeth Keddie-Gray, Natalie Keenan, Jonathan Kefas, Stephen Kegg, Laura Keith, Uzoamaka Keke, Joanne Kellett, Alison Kelly, David Kelly, Diane Kelly, Dominic Kelly, Emma Kelly, Laura Kelly, Martin Kelly, Michael Kelly, Rosalind Kelly, Sinead Kelly, Stephen Kelly, Stephen Kelly, Mary Kelly-Baxter, Marketa Keltos, Timothy Kemp, Kelly Kemsley, Alexandra Kendall-Smith, Sarah Kennard, Ann Kennedy, James Kennedy, Sophie Kennedy-Hay, Julia Kenny, Melanie Kent, Lynne Keogan, Alexander Keough, Andrew Kerr, Caroline Kerrison, Anthony Kerry, Sam Kershaw, Helen Kerslake, Ian Kerslake, Helen Kerss, Jocelyn Keshet-Price, Georgina Keyte, Abdul Khadar, Ali Khalid, Muhammad U Khalid, Syed Khalid, Amir Khalil, Asma Khalil, Sijjad Khalil, Abubakar Khan, Ali Khan, Al-Imran Khan, Arham Khan, Asad Khan, Aurangzeb Khan, Burhan Khan, Camran Khan, Fatimah Khan, Kausik Khan, Malik A Khan, Marria Khan, Mehrunnisha Khan, Mohammad Khan, Nayeem Khan, Omar Khan, Rahila Khan, Shabana Khan, Shahul Khan, Shoaib Khan, Tasaduksultan Khan, Waseem Khan, Usman F Khatana, Jibran Khatri, Jyoti Khatri, Hafiza Khatun, Taslima Khatun, Mena Kheia, Jacyntha Khera, Htet H E Khin, Najaf Khoja, Kiran Khokhar, Chloe Khurana, Faith Kibutu, Andrew damian, Michelle Kidd, Joe Kidney, Shane Kidney, Will Kieffer, James Kilbane, Caroline Kilby, Eileen Killen, Susan Kilroy, Bomee Kim, Jee Whang Kim, Sarah Kimber, Andy King, Barbara King, Jennifer King, Kirsten King, Rachel King, Sarah King, Victoria King, Emily King-Oakley, Laura Kingsmore, Fiona Kinney, Sidra Kiran, Jeremy Kirk, Jodie Kirk, Amy Kirkby, Emily Kirkham, Gemma Kirkman, Ursula Kirwan, Kelly Kislingbury, Toby Kitching, Laura Kitto, Lauren Kittridge, Sarah Klaczek, Frieder Kleemann, Susan Kmachia, Chris Knapp, Lucy Knibbs, Alicia Knight, Fraser Knight, Marian Knight, Sarah Knight, Steven Knight, Tom Knight, Ellen Knights, Jane Knights, Martin Knolle, Carol Knott, Charlotte Knowles, Karen Knowles, Karen Knowles, Laurence Knowles, Emily Knox, Lucy Knox, Oliver Koch, Ronan Kodituwakku, Gouri Koduri, Aisha Koirata, Eirene Kolakaluri, Magdalena Kolodziej, Eirini Kolokouri, Samantha Kon, Niladri Konar, Mari Kononen, Athanasios Konstantinidis, Hui Fen Koo, Imogen Koopmans, Emmanuela Kopyj, Laura Korcierz, James Korolewicz, George Koshy, Chris Kosmidis, Jalpa Kotecha, Easwari Kothandaraman, Koushan Kouranloo, Rukhsana Kousar, Margarita Kousteni, Maja Kovac, Alex Kozak Eskenazia, Kestutis Krasauskas, Raghu Krishnamurthy, Vinodh Krishnamurthy, Manju Krishnan, Hari Krishnan, Suzanne Krizak, Sean Krupej, Agnieszka Kubisz-Pudelko, Soren Kudsk-Iversen, Aurimas Kudzinskas, Chirag Kukadiya, Nainesha Kulkarni, Aditi Kumar, Mayur Kumar, Ramesh Kumar, Ravi Kumar, Rita Kumar, Rupa Kumar, Satish Kumar, Vimal Kumar, Arun Kundu, Heinke Kunst, Amit Kurani, Mohammed Kurdy, Rincy Kurian, Vimal Kurmars, Cameron Kuronen-Stewart, Ranganai S Kusangaya, Vlad Kushakovsky, Mandy Kuunal, Apexa Kuverji, Amma Kyei-Mensah, Thyra Kyere-Diabour, Moe Kyi, Nyan M Kyi, Laura Kyle, Karali-Tsilimpari Kyriaki, Julius Labao, Louise Lacey, Nikki Lack, Emma Ladlow, Heather Lafferty, Shondipon Laha, Sushil Lahane, Clement Lai, James Lai, Robert Laing, Inez Laing-Faiers, Emily Laity, Nicki Lakeman, David Lalloo, Fiona Lalloo, Alison Lam, Fiona Lamb, Lucy Lamb, Thomas Lamb, Pauline Lambert, Claudia Lameirinhas, Mohammed K G Lami, Holly Lamont, Michal Lamparski, Djillali Lamrani, Christine Lanaghan, Ivone Lancona-Malcolm, Julia Lancut, Geraldine Landers, Martin J Landray, Matthew Lane, Nicholas Lane, Alidih Lang, Stephen Lang, Daniel Langer, Margaret Langley, Charles Langoya, Emily Langthorne, Taiya Large, Anna Last, Scott Latham, John Latham-Mollart, Afzal Latheef, Nang Latt, Carly Lattimore, Dawn Lau, Eva Lau, Myra Laurenson, Hou Law, Jennifer Law, Jessica Law, Penny Law, Richard Law, Emma Lawrence, Neil Lawrence, Ryan Lawrie, Joanne Lawson, Louise Lawson, Michael Lay, Christine Laycock, Reina Layug, Maria Lazo, Vietland Le, Amelia Lea, William Lea, Ian Leadbitter, Thomas Leahy, Richard Lean, Lorna Leandro, Darren Leaning, Sandra Leason, Emma Lee, Hannah Lee, Irish Lee, Judith Lee, Sam Lee, Shi H Lee, Simon Lee, Sindy Lee, Stephanie Lee, Tracey Lee, Xiang Lee, Diana Lees, Jennifer Lees, Helen Legge, Julian Leggett, Katie Leigh-Ellis, Kevan Leighton, Nicky Leitch, Eleni Lekoudis, Petula Lemessy, Nicholas Lemoine, Katy Leng, Katrina Lennon, Liz Lennon, Kelly Leonard, Wen Leong, Nicky Leopold, Oskar Lepiarczyk, Isla Leslie, Eleni Lester, Joe Leung, Emma Levell, Chris Levett, Alice Lewin, Michaela Lewin, Alison Lewis, David Lewis, Dee Lewis, Gillian Lewis, Joanne Lewis, Joseph Lewis, Kathryn Lewis, Keir Lewis, Leon Lewis, Marissa Lewis, Rob Lewis, Robert Lewis, Catherine Lewis-Clarke, Katherine Lewiston, Adam Lewszuk, Penny Lewthwaite, Samantha Ley, Angela Liao, Victoria Licence, David Lieberman, Susan Liebeschuetz, Nicky Lightfoot, Patrick Lillie, Ben Lim, Carys Lim, Ee Thong Lim, Ivy Lim, Terence Lim, Wei Shen Lim, Wilson Lim, James Limb, Usha Limbu, Christian Linares, Dermot Linden, Gabriella Lindergard, Kate Lindley, Charlotte Lindsay, Emily Lindsay, Max Lindsay, Helen Lindsay- Clarke, Mirella Ling, Claire Lingam, Linette Linkson, Mike Linney, Louise Linsell, Conrad Lippold, George Lipscomb, Karen Lipscomb, Laura Lipskis, Ana Lisboa, Evangeline Lister, Jeff Little, Sam Little, Xuedi Liu, Daniel Kevin Llanera, Rhiannon Llewellyn, Martin Llewelyn, Adam Lloyd, Aimee Lloyd, Arwel Lloyd, Oliver Lloyd, Richard Lloyd, Su Lo, David Loader, Lydianne Lock, Sara Lock, Stephen Lock, Angela Locke, Jacqueline Locke, Thomas Locke, Teresa Lockett, Jeorghino Lodge, Terrence Lodge, Martina Lofthouse, Heather Loftus, Meg Logan, Chloe Logue, Sook Y Loh, Siddharth Lokanathan, Kaatje Lomme, Emily London, Gabriella Long, Natalie Long, Bev Longhurst, Mark Longshaw, Jennifer Lonnen, Caroline Lonsdale, Laura Looby, Ronda Loosley, Paola Lopez, Paula Lopez, Robert Lord, Catherine Lorenzen, Claire Lorimer, Francesco Loro, Rachel Lorusso, Robert Loveless, Maxine Lovell, Angeliki Loverdou, Andrew Low, Jen M Low, Alastair Lowe, Caroline Lowe, Catherine Lowe, Emily Lowe, Faye Lowe, Michael Lowe, Richard Lowsby, Vicki Lowthorpe, Gamu Lubimbi, Alexandra Lubina Solomon, Georgia Lucas, Jacob Lucas, Alice Lucey, Olivia Lucey, Suzanne Luck, Jane Luke, Apurva Lunia, Muriel Lunn, Ji Luo, Cindy Nisha Luximon, Barrie Lyell, Elisavet Lyka, Audrey Lynas, Ceri Lynch, Daniel Lynch, Daniella Lynch, Stephen Lynch, Helen Lyon, Rea-Grace Maamari, Hannah Mabb, Louies Mabelin, Jessica Macaro, Kateryna Macconaill, Chloe Macdonald, Stuart MacDonald, Claire Macfadyen, James Gray Macfarlane, Jill Macfarlane, Laura Macfarlane, Lisa MacInnes, Iain MacIntyre, Jill MacIntyre, Kirsten Mack, Callum Mackay, Euan Mackay, Laura Mackay, Alexander Mackenzie, Matt Mackenzie, Robert MacKenzie Ross, Ami Mackey, Fiona Mackie, Robert Mackie, Carolyn Mackinlay, Claire Mackintosh, Katherine Mackintosh, Mary Joan MacLeod, Michael Macmahon, Andrew MacNair, Catherine Macphee, Iain Macpherson, Catriona Macrae, Allan MacRaild, Alannah Madden, Mary Madden, Norman Madeja, Karen Madgwick, Pradeep Madhivathanan, Madhavi Madhusudhana, Alpha Madu, Lorraine Madziva, Marion Mafham, Nick Magee, Frederick Magezi, Negar Maghsoodi, Christopher Magier, Marios Magriplis, Natasha Mahabir, Subramanian Mahadevan-Bava, Anjanie Maharajh, Ajit Mahaveer, Bal Mahay, Kanta Mahay, Hibo Mahdi, Thushika Mahendiran, Siva Mahendran, Sarah Maher, Anistta Maheswaran, Shameera Maheswaran, Tina Maheswaran, Parisa Mahjoob-Afag, Ahmed Mahmood, Farhana Mahmood, Waheed Mahmood, Zahra Mahmood, Hager Mahmoud, Ewan Mahony, Luke Mair, Toluwani Majekdunmi, Kesson Majid, Rupert Major, Jaydip Majumdar, Mohammad K H Majumder, Stephen Makin, Marius Malanca, Hannah Malcolm, Flora Malein, Neeraj Malhan, Ayesha Malik, Gulshan Malik, Mohammed Maljk, Paul Mallett, Petrina Mallinder, Georgia Mallison, Louise Mallon, Edward Malone, Gracie Maloney, Edgar Malundas, Madhu Mamman, Irene Man, Kathy Man, Rossana Mancinelli, Marco Mancuso-Marcello, Tracy Manders, Lauren Manderson, Justin Mandeville, Roope Manhas, Carmen Maniero, Ravi Manikonda, Bobby Mann, Jonathan Manning, Katherine Mansi, Katarina Manso, Dina Mansour, Isheunesu T Mapfunde, Predeesh Mappa, Hemant Maraj, Clare Marchand, Neil Marcus, Maria Marecka, Gomathi Margabanthu, Jordi Margalef, Lavinia Margarit, Georgios Margaritopoulos, Mike Margarson, Fernandez M Maria del Rocio, Teresa Maria Pfyl, Victor Mariano, Helen Maria-Osborn, Ashleigh Maric, Grace Markham, Maria Marks, Pamela Marks, Elisabeth Marouzet, Arran Marriott, Cheryl Marriott, Nemonie Marriott, Karen Marsden, Paul Marsden, Sarah Marsden, Tracy Marsden, Robyn Marsh, Adam Marshall, Andrew Marshall, Gail Marshall, Henry Marshall, Jaimie Marshall, Jenna Marshall, Nicola Marshall, Riley Marshall, Jennifer Marshall, Samantha Marston, Emmeline Martin, Hayley Martin, Hope Martin, Jane Martin, Karen Martin, Kate Martin, Laila Martin, Michael Martin, Noelia Martin, Tim Martin, Winston Martin, Tim Martindale, Marcus Martineau, Lauren Martinez, Jose C Martinez Garrido, Juan Martin-Lazaro, Vijay K Maruthamuthu, Gemma Maryan, Roman Mary-Genetu, Sam Maryosh, Vidan Masani, Diego Maseda, Sheila Mashate, Yasaman Mashhoudi, Al Mashta, Izhaq Masih, Sanna Masih, Nick Maskell, Nick Maskell, Perry Maskell, Matthew Masoli, Rebecca Mason, Richard Mason, Ruth Mason, Claire Mason, Mohammad Masood, Mohammad T Masood, Syed Masood, Syed S M E Masood, Aaqib Masud, Lear Matapure, Cristina Matei, Ropafadzo Matewe, Manraj Matharu, Stephy Mathen, Alex Mather, Nicole Mather, Jonathan Mathers, Joanna Matheson, Amal Mathew, Anna Mathew, Moncy Mathew, Verghese Mathew, Caroline Mathews, Jesha Mathews, Kate Mathias, Darwin Matila, Wadzanai Matimba-Mupaya, Nashaba Matin, Elina Matisa, Max Matonhodze, Elijah Matovu, Jaysankar Mattappillil, Alison J Matthews, Heather Matthews, Helen Matthews, Gwynn Matthias, Fiona Maxton, Adam Maxwell, Veronica Maxwell, James May, Joanne May, Philippa May, Irving Mayanagao, Matthew Maycock, Graham Mayers, Shelley Mayor, Ibreaheim Mazen, Andrea Mazzella, Nyambura Mburu, Eleanor McAleese, Helinor McAleese, Paul McAlinden, Audrey McAlpine, Graeme McAlpine, Jonathan McAndrew, Hamish McAuley, Sarah McAuliffe, Claire McBrearty, Erin McBride, Michael McBuigan, James McBurney, Laura McCabe, Amanda McCairn, Jake McCammon, Nicole McCammon, Conor McCann, Alexandra McCarrick, Brendan McCarron, Eoghan McCarthy, Michelle McCarthy, Natalie McCarthy, Sinead McCaughey, Gareth McChlery, Tara McClay, Beverley McClelland, Declan McClintock, Patricia McCormack, Jacqueline McCormick, Wendy McCormick, Paul McCourt, Jame McCrae, Sharon McCready, Gordan McCreath, Helen McCreedy, Louise McCreery, Iain J McCullagh, Liz McCullagh, Megan McCullagh, Conor McCullough, Katherine McCullough, Nicola McCullough, Sarah McCullough, Fiona McCurrach, Rory McDermott, Katharine McDevitt, Helen McDill, Basil McDonald, Claire McDonald, Debbie McDonald, Rob McDonald, Sam McDonald, Damhnaic McDonald, Rowan McDougall, Irene McEleavy, Julie McEntee, Evanna McEvoy, Ruth McEwen, Margaret McFadden, Denise McFarland, Margaret McFarland, Rachel McFarland, Erin McGarry, Lorcan McGarvey, Clodagh McGettigan, Michael McGettrick, Christopher McGhee, Fiona McGill, Sarah McGinnity, Neil McGlinchey, Phil McGlone, Deborah McGlynn, Claire McGoldrick, Clare McGoldrick, Elizabeth McGough, Brendan McGrath, Amanda McGregor, Annemarie McGregor, Cathryn McGuinness, Heather McGuinness, Sean McGuire, Tara McHugh, Caroline McInnes, Neil McInnes, Karen McIntyre, Mhairi McIntyre, Lorna McKay, Conor P McKeag, Madeleine McKee, Joseph McKeever, Shirley McKenna, Donogh McKeogh, Caroline McKerr, Anthony M McKie, Laura Mckie, Gerard McKnight, Heather McLachlan, Andrew McLaren, Barbara McLaren, Nicola McLarty, Maria McLaughlin, James McLay, Mary McLeish, Tina McLennan, Stewart McLure, Anne M McMahon, Genevieve McMahon, Mike McMahon, Stephen McMahon, Terence McManus, Moyra McMaster, Paddy McMaster, Samuel McMeekin, Nicola McMillan, Jason McMinn, Liam McMorrow, Helen McNally, Fiona McNeela, Lynne McNeil, Claire McNeill, Shea McNeill, Una McNelis, Melanie McNulty, Roisin McNulty, Christopher McParland, Mark McPhail, Alison McQueen, Anna McSkeane, Denise McSorland, Gini McTaggart, Jacqueline McTaggart, Joanna Mead, Emma Meadows, Olivia Meakin, Ben Mearns, Claire Mearns, Kim Mears, William Mears, Manjula Meda, Ayren Mediana, Ross Medine, Thomas Medveczky, Sharon Meehan, Emily Meeks, Abbi Megan, Nevan Meghani, Salim Meghjee, Rohan Mehra, James Meiring, Rayane Mejri, Sabina Melander, Adriana-Stefania Melinte, Francesca Mellor, Samantha Mellor, Zoe Mellor, Katrina Mellows, Vladimir Melnic, Alice Melville, Julie Melville, Helen Membrey, Mark Mencias, Cheryl Mendonca, Alexander Mentzer, Dan Menzies, Sue Mepham, Oliver Mercer, Pauline Mercer, Arwa Merchant, Fatema Merchant, Mihaela Mercioniu, Megan Meredith, Marta Merida Morillas, Blair Merrick, Jack Merritt, Simon Merritt, Ekta Merwaha, Simon Message, Gabriel Metcalf-Cuenca, Benjamin Metcalfe, Kneale Metcalfe, Stella Metherell, Alexsandra Metryka, Louise Mew, Simon Meyrick, Nhlanhla Mguni, Atiqa Miah, Jagrul Miah, Nahima Miah, Gabriela Mic, Dariush Micallef, Alice Michael, Angiy Michael, Shery Michael, Vincent Michael, Natalia Michalak, Loredana Michalca-Mason, Janet Middle, Hayley Middleton, Jennifer T Middleton, Maeve Middleton, Sophie Middleton, Shelley Mieres, Loredana Mihalca-Mason, Theresia Mikolasch, Sarah Milgate, Colin Millar, Jonathan Millar, David Miller, Johnathan Miller, Lucy Miller, Rachel Miller, Naomi Miller-Biot, Alex Miller-Fik, Louise Millett, Hazel Milligan, Iain Milligan, Caitlin Milliken, Katherine Millington, Samuel Millington, Helen Mills, Janet Mills, Helen Millward, Rebecca Miln, Alice Milne, Charlotte Milne, Louise Milne, Joanne Milner, Zayar Min, Samuel Mindel, Chrissie Minnis, Paul Minnis, Jane Minton, Frederico Miranda, Lucy Mires, Taimur Mirza, Anjum Misbahuddin, Aseem Mishra, Biswa Mishra, Eleanor Mishra, Ritu Mishra, Sannidhya Misra, Deena Mistry, Heena Mistry, Dushyant Mital, Sarah Mitchard, Alan Mitchell, Ben Mitchell, Piers Mitchell, Susan Mitchell, Philip Mitchelmore, Andrew Mitra, Atideb Mitra, Sandip Mitra, Clarisse Mizzi, Emma Moakes, Emma Moatt, Gita Modgil, Abdelrahman Mohamed, Arez Mohamed, Osab Mohamed, Waheed Mohammad, Aliabdulla Mohammed, Omer Mohammed, Yaser N S Mohammed, Bilal A Mohamud, Mahalakshmi Mohan, Amr Moharram, Jonathan Mok, Christine Moller-Christensen, Mateus Mollet, Malid Molloholli, Aoife Molloy, Linda Molloy, Andrew Molyneux, Tasnim Momoniat, Holly Monaghan, Krista Monaghan, Shiva Mongolu, Katelyn Monsell, Mahmoud Montasser, Alan Montgomery, Hugh Montgomery, Prebashan Moodley, Margaret Moody, Nick Moody, Angela Moon, James Moon, Ji-Hye Moon, Maria Moon, May Moonan, Parvez Moondi, Alex Moore, Christopher Moore, David Moore, Faye Moore, Judith Moore, Laura Moore, Sally Moore, Sonia Moore, Rachel Moores, Ed Morab, Jose Morales, Nuria Moramorell, Louise Moran, Grishma Moray, Jeronimo Moreno-Cuesta, Amy Morgan, Christine Morgan, Colin Morgan, Lauren Morgan, Leila Morgan, Matthew Morgan, Patrick Morgan, Katie Morgan-Jones, Emily Morgan-Smith, Anna Morley, Thomas Morley, Wendy Morley, Anna Morris, Damian Morris, Fiona Morris, Helen Morris, Juliet Morris, Katie Morris, Laura Morris, Lucy Morris, Mary-Anne Morris, Niall Morris, Paul Morris, Sheila Morris, Susan Morris, Douglas Morrison, Moira Morrison, Mary Morrissey, Anna Morrow, Franca Morselli, Gordon Mortem, Chelsea Morton, Gordon Morton, Priti Morzaria, Alison Moss, Charlotte Moss, Sarah Moss, Stuart Moss, Nicki Motherwell, Johanna Mouland, Caroline Moulds, Hilary Moulton, Elizabeth Mousley, Karen Moxham, Borja Moya, Quberkani Moyo, Eunice Mshengu, Sheila Mtuwa, Ali Muazzam, Iqtedar A Muazzam, Nykki Muchenje, Dalia Mudawi, Girish Muddegowda, Imran Mugal, Ahsan Mughal, Javaid Muglu, Javed Muhammad, Alison Muir, Carol Muir, Martin Muir, Dipak Mukherjee, Syed A A Mukhtar, Denise Mukimbiri, Peter Mulgrew, Ben Mulhearn, Arafat Mulla, Dee Mullan, Dileepkumar Mullasseril Kutten, Niall Mullen, Rosemary Mullett, Sandra Mulligan, Lana Mumelj, Andrew Mumford, Mohammed Munavvar, Henry Munby, Anne-Marie Munro, Sheila Munt, McDonald Mupudzi, Arshid Murad, Oluwatosin H Muraina, Koteshwara Muralidhara, Diane Murdoch, Mhairi Murdoch, Jennifer Murira, Alison Murphy, Carl Murphy, Gail Murphy, Peter Murphy, Sheenagh Murphy, Simon Murphy, Clare Murray, David Murray, Eleanor Murray, Katie Murray, Kenneth Murray, Lisa Murray, Lorna Murray, Tracey Murray, Eoin Murtagh, Mithun Murthy, Catherine Murton, Rosie Murton, Neeka Muru, Rosemary Musanhu, Maimuna Mushabe, Omaisa Mushtaq, Ahmed M M Mustafa, Elhaytham Mustafa, Mustafa Mustafa, Ibrahim Mustapha, Zhain Mustufvi, Callum Mutch, Eric Mutema, Balakumar Muthukrishnan, Sheree Mutton, Natasha Muzengi, Memory Mwadeyi, Bettina Mwale, Esther Mwaura, Raji Myagerimath, Alice Myers, Sam Myers, Khin S Myint, Yadee Myint, Libor Myslivecek, Helen Nabakka, Evelyn Nadar, Iftikhar Nadeem, Moosa Nadheem, Asma Naeem, Hassan Naeem, Salman Naeem, Samraiz Nafees, Mohamed Nafei, Thapas Nagarajan, Imrun Nagra, Deepak Nagra, Mina Naguib, Kirushthiga Naguleswaran, K Shonit Nagumantry, Kevin Naicker, Sarveshni Naidoo, Gireesha Naik, Rishi Naik, Samir Naik, Devu Sasikumar Nair, Rajiv Nair, Tanushree Nair, Jay Naisbitt, Kerry Naismith, Sri Nallapareddy, Soum Nallapeta, Arumugan Nallasivan, Uttam Nanda, Aarti Nandani, Ali R Naqvi, Asadullah Naqvi, Sara Naqvi, Sophia Nasa, Dominic Nash, Nader Nasheed, Abdul Nasimudeen, Umer Nasir, Marwan Nassari, Tahir Nasser, Anuja Natarajan, Geetha Natarajan, Nalin Natarajan, Nikhila Natarajan, Rajkumar Natarajan, Noel Nathaniel, Mala Nathvani, Priyan Nathwani, George Nava, Neena Navaneetham, Jeya Navaratnam, Helen Navarra, Sadaf Naveed, John Navin, Khuteja Nawaz, Sarfaraz Nawaz, Shasta Nawaz, Bonilla Nayar, Suzanne Naylor, Moez Nayyar, Farrah Naz, Mobeena Naz, Babak Nazari, Sehar Nazir, Dumisani Ncomanzi, Onyine Ndefo, Alan Neal, Elaine Neary, Mostafa Negmeldin, Paula Neill, Hector E Neils, Avideah Nejad, Louise Nel, Marie Nelson, Richard Nelson, Scott Nelson, Rajesh Nemane, Samiksha Nepal, Daniel Nethercott, Kimberley Netherton, Kimberley Nettleton, Alison Newby, Angela Newby, David Newby, Tracy Newcombe, Charlotte Newman, Diana Newman, Julie Newman, Oscar Newman, Tabitha Newman, Thomas Newman, Rachel Newport, Christopher Newson, Maria Newton, Anthony Y K C Ng, Ka Wing Ng, Maxine Ng, Sarah Ng, Wee Jin Ng, Thomas Ngan, Gabriel CE Ngui, Alice Ngumo, Caoimhe Nic Fhogartaigh, Nathalie Nicholas, Philip Nicholas, Rachel Nicholas, Donna Nicholls, Lisa Nicholls, Alice Nicholson, Anne Nicholson, Annette Nicholson, Ian Nickson, Eileen Nicol, Elizabeth Nicol, Rebecca Nicol, Pantelis Nicola, Antony Nicoll, Pantzaris Nikolaos, Georgii Nikonovich, Annette Nilsson, Kofi Nimako, Louise Nimako, Camus Nimmo, Preethy Ninan, Mahesh Nirmalan, Muhammad Nisar, Toby Nisbett, Aksinya Nisha James, Sabaahat Nishat, Tomoko Nishiyama, Sara Nix, Jennifer Nixon, Maxine Nixon, Khwaja Nizam Ud Din, Maria Nizami, Lyrics Noba, Harriet Noble, Hsu Noe, Jerry Nolan, Zahid Noor, Zaid Noori, Louis Norman, Rachel Norman, Karen Norris, Lillian Norris, Sally Ann Nortcliffe, Fiona North, Julie North, Thomas North, John Northfield, Samantha Northover, Jurgens Nortje, Donna Norton, Rowen Norton, Holly Notman, Khalid Nourein, Timea Novak, Alan Noyon, Arlene Nubi, Mohamed Nugdallah, Anne Marie Nugent, Justine Nugent, Kribashnie Nundlall, Kieran Nunn, Michelle Nunn, Jane Nunnick, Yvonne Nupa, Zubeir Nurgat, Godfrey Nyamugunduru, Maggie Nyirenda, Kerry Nyland, Daire O Shea, Sara O'Brien, Ruth O'Donnell, Chloe O'Hara, Kevin O'Reilly, Caroline Oakley, Begho Obale, Clements Oboh, Clare O'Brien, Julie O'Brien, Kirsty O'Brien, Linda O'Brien, Marese O'Brien, Neale O'Brien, Rachel O'Brien, Tracey O'Brien, Emma O'Bryan, Ross Obukofe, Christopher O'Callaghan, Lorcan O'Connell, Tadg O'Connor, Chris O'Connor, Grainne O'Connor, Miranda Odam, Sam Oddie, Sharon Oddy, Yejide Odedina, Krishma Odedra, Sven Wilhelm Odelberg, Natasha Odell, Omolola Oderinde, Jessica Odone, Catherine O'Donovan, Stephen O'Farrell, Pamela Offord, Tanwa Ogbara, Catherine Ogilvie, Ciaran O'Gorman, Oluwatomilola Ogunkeye, Udeme Ohia, Shinjali Ohja, Ohiowele Ojo, Mark O'Kane, Tolu Okeke, Eleanor OKell, Alicia Okines, Iheoma Okpala, Ernest Okpo, Maryanne Okubanjo, Raphael Olaiya, Tim Old, Jane Oldham, Gregory Oleszkiewicz, Annie Oliver, Catherine Oliver, Jesse Oliver, Martyn Oliver, Zoe Oliver, Nurudeen O Olokoto, Folusho Olonipile, Olumide Olufuwa, Olatomiwa Olukoya, Akinlolu Oluwole-Ojo, Laura O'Malley, Maryam Omar, Zohra Omar, Nimca Omer, Connaire O'Neill, Lauran O'Neill, Chon Sum Ong, Chidera Onyeagor, Huah Chiang Ooi, Amin Oomatia, Maria Opena, Richard Oram, Christy Ord, Jonathan Ord, Lola Orekoya, Devaki O'Riordan, Sean O'Riordan, Amy Orme, Hannah Orme, Charlotte Orr, Sarah Orr, Christopher Orton, Anna Osadcow, Rawlings Osagie, Rostam Osanlou, Lynn Osborne, Nigel Osborne, Rebecca Osborne, Wendy Osborne, William Osborne, Charles Osbourne, Jennifer Osei-Bobie, Mandy O'Shea, Joseph Osman, Wa'el Osman, Bashir Osman, G Osoata, Marlies Ostermann, Eoin O'Sullivan, Susan O'Sullivan, Noor Otey, Otheroro K Otite, Marie O'Toole, Rachel Owen, Stephanie Owen, Emma Owens, Yetunde Owoseni, Michael Owston, Ruth Oxlade, Feray Ozdes, Jamie Pack, Sophie Packham, Piotr Paczko, Grace Padden, Anand Padmakumar, Iain Page, Valerie Page, Jodi Paget, Katherine Pagett, Lee Paisley, Susie Pajak, Angela Pakozdi, Soubhik Pal, Sushi Pal, April Palacios, Vishnu B Palagiri Sai, Vadivu Palaniappan, Priya Palanivelu, Adrian Palfreeman, Deepshikha Palit, Alistair Palmer, Lynne Palmer, Ian Pamphlett, Anmol Pandey, Nithya Pandian, Krishnaa Pandya, Tej Pandya, Alice Panes, Yee Wei Pang, Laura Pannell, Kanwar Pannu, Sathianathan Panthakalam, Charles T Pantin, Norman Pao, Helen Papaconstantinou, Padmasayee Papineni, Kitty Paques, Kerry Paradowski, Vinay Parambil, Supathum Paranamana, Siddhant Parashar, Ian Parberry, Amy Parekh, Dhruv Parekh, Louise Parfitt, Helen Parfrey, Omi Parikh, Gemma Parish, John Park, Angela Parker, Ben Parker, Emma Parker, Fiona Parker, Jacob Parker, Julie Parker, Laura Parker, Lucy Parker, Sara Parker, Sean Parker, Kirstin Parkin, Anna Parkinson, Valerie Parkinson, Chetan Parmar, Viraj Parmar, Victoria Parris, Helen C Parry, Siobhan Parslow-Williams, Maria Parsonage, Penny Parsons, Sarah Parsons, Richard Partridge, Kevin Parvin, Lauren Passby, Juan Pastrana, Mital Patal, Sarah Patch, Aamie Patel, Alkesh Patel, Amisha Patel, Dakshesh Patel, Darshna Patel, Hemani Patel, Jaymik Patel, Kamal Patel, Kayur Patel, Kiran Patel, Krish Patel, Manish Patel, Martyn Patel, Mehul Patel, Naleem Patel, Nehalbhai Patel, Prital Patel, Rebecca Patel, Saagar Patel, Soonie Patel, Trishna Patel, Vishal Patel, Sangeeta Pathak, Nazima Pathan, Alexandra Patience, Donna Patience, Abigail Patrick, Georgie Patrick, Jean Patrick, Simon Patten, Ben Pattenden, Charlotte Patterson, Linda Patterson, Molly Patterson, Rob Patterson, Robert Patterson, Leigh Pauls, Stephane Paulus, Amelia Pavely, Susan Pavord, Brendan Payne, David Payne, Elizabeth Payne, Ruth Payne, Tammy Payne, Linda Peacock, Louise Peacock, Sarah Peacock, Henry Peake, Rupert Pearse, Andrew Pearson, Daniel Pearson, Harriet Pearson, Karen Pearson, Kirsty Pearson, Samuel A Pearson, Sandra Pearson, Alice Peasley, Hilary Peddie, Russell Peek, Claire Pegg, Suzannah Peglar, Benjamin H Peirce, Claire Pelham, Abigail Pemberton, Melchizedek Penacerrada, Anthony Pender, Carmel Pendlebury, Jessica Pendlebury, Rachel Penfold, Catherine Penman, Julie Penman, Rachel Penman, Justin Penner, Kristi Penney, Alistair Penny, James Penny, Justin Pepperell, Huw Peregrine, Adriana Pereira, Adriana Pereira, Rita Pereira, Carlota Pereira Dias Alves, Elena Perez, Jane Perez, Tanaraj Perinpanathan, Lakshmi Periyasamy, Francesca Perkins, Elizabeth Perritt, Alison Perry, Emily Perry, Meghan Perry, Terrie Perry, Thomas M Perumpral, Guilherme Pessoa-Amorim, Ruth Petch, Lionel Peter, Cecilia Peters, Mark Peters, Steve Peters, Tim Peters, Remy Petersen, Alexandra Peterson, Leon Peto, Iulia Petras, Ilianna Petrou, Boyanka Petrova, Mirela Petrova, Paul Pfeffer, Mysore Phanish, Paul Phelan, Christopher Philbey, Jennifer Philbin, Alex Phillips, Dylan Phillips, Karen Phillips, Rachael Phillips, Marie Phipps, Virach Phongsathorn, Mandeep Phull, Masroor M Phulpoto, Myat T T PI, Sara Pick, James Pickard, Charlotte Pickering, Gillian Pickering, Thomas Pickett, Joanna Pickles, Benjamin Pickwell-Smith, Natalia Pieniazek, Charlie Piercy, Angelo Pieris, Samia Pilgrim, Paul A Pillai, Zoe Pilsworth, Heather Pinches, Stacey Pinches, Kirsty Pine, Muni T Pinjala, Stefania Pintus, Graeme Piper, Tasneem Pirani, Marcus Pittman, Sally Pitts, Nicolene Plaatjies, Aiden J Plant, Naomi Platt, Robert Pleass, Laura Plummer, Charles Plumptre, Jonathan Pobjoy, Tatiana Pogreban, Stephen Poku, Rachel Pollard, Louisa Pollock, Louisa Pollock, Oluwamayowa Poluyi, Gary J Polwarth, Fiona Pomery, Ponmurugan Ponnusamy, Suresh Ponnusamy, Aravind Ponnuswamy, Inês Ponte Bettencourt dos Reis, Suman Pooboni, Alice Poole, Christopher Poole, Lorraine Poole, Lynda Poole, Michele Poole, Sharon Poon, Tajinder Poonian, David Porter, Jo Porter, Linda Porter, Ross Porter, Kelly Postlethwaite, Narayana Pothina, Priyadarshan Potla, Dorota Potoczna, Jason Pott, Alison Potter, Jean Potter, Sarah Potter, Tracey Potter, Elspeth Potton, Joanne B Potts, Julie Potts, Kathryn Potts, K Poultney, Una Poultney, Vanessa Poustie, James Powell, Jordan Powell, Deborah Power, Nick Power, Joseph Poxon, Robin Poyner, Vidushi Pradhan, Helena Prady, Aalekh Prasad, Krishna Prasad, Fredy Prasanth Raj, Sangeetha Prasath, Anezka Pratley, Steven Pratt, David Preiss, Claire Prendergast, Lynn Prentice, Peter Prentice, Verity Prescott, Laura Presland, Catharine Prest, Stephen Preston, Martha Pretorius, Natalie Prevatt, Sandra Prew, Ashley Price, Carly Price, Claire Price, David Price, Elizabeth Price, Nathan Price, Vivien Price, Anne Priest, Kate Priestley, Jimena Prieto, Lorraine Primrose, Clare Prince, Judith Prince, Laura Prince, Shirley Pringle, Veronika Pristopan, Kelly Pritchard, Lucy Pritchard, Rhys Pritchard, Simon Pritchard, Verma Priyash, Andrew Procter, Clare Proctor, Rebecca Proudfoot, Ben Prudon, David Pryor, Solomon Pudi, Joanne Pugh, Lawrence Pugh, Mark T Pugh, Nichola Pugh, Richard Pugh, Veronika Puisa, Kirandip Punia, Saleel Punnilath Abdulsamad, Laura Purandare, Corrina Purdue, Bally Purewal, Molly Pursell, Gregory Purssord, Sarah Purvis, Kathryn Puxty, Zoe Puyrigaud, Michael Pynn, Tariq Qadeer, Mohammad Qayum, Corrine Quah, Sheena Quaid, Nathaniel Quail, Charlotte Quamina, Alice Quayle, Eleanor Quek, Siobhan Quenby, Xinyi Qui, Vanessa Quick, Julie Quigley, Juan-Carlos Quijano-Campos, Andrew Quinn, Tom Quinn, Quratulain Quratulain, Danya Qureshi, Ehsaan Qureshi, Hasanain Qureshi, Khadija Qureshi, Nawaz Qureshi, Qurratulain Qurratulain, Saad Qutab, Muhammad S Rabbani, Simon Rabinowicz, Madalina Raceala, Raissa Rachman, Laura Rad, Jane Radford, Liz Radford, Jayachandran Radhakrishnan, Cecillia Rafique, Jethin Rafique, Muhammad Rafique, Ravi Ragatha, Aiswarya Raghunathan, Abigail Raguro, Shankho D Raha, Sana Rahama, Karen Rahilly, Faisal Rahim, Abdul H Rahimi, Haseena R Rahimi, Muhammad Rahman, Salim Ur Rahman, Lenka Raisova, Arjun Raj, Pradeep Rajagopalan, Nithy Rajaiah, Arvind Rajasekaran, Aylur Rajasri, Thurkka Rajeswaran, Jyothi Rajeswary, Jeyanthy Rajkanna, Gayathri Rajmohan, Ruth Rallan, Katherine Ralston, Maximilian Ralston, Matsa Ram, Balaji Ramabhadran, Fathima Ramali, Mohamed Ramali, Athimalaipet Ramanan, Shashikira Ramanna, Maheshi Ramasamy, Jozel Ramirez, Mylah Ramirez, Geshwin Ramnarain, Lidia Ramos, Shanthi Ramraj, Alex Ramshaw, Aleem Rana, Ghulam F Rana, Rehman Rana, Abby Rand, James Rand, Harpal Randheva, Poonam Ranga, Manmeet Rangar, Harini Rangarajan, Sameer Ranjan, Poormina Ranka, Rajesh Rankhelawon, Anita Rao, Sandhya Rao, Sanjay Rao, Deepak Rao, Anuja Rasarathnam, Althaf A Rasheed, Khalid Rashid, Simbisai Ratcliff, Sam Ratcliffe, Sophy Ratcliffe, Sanjeev Rath, Mohmad I Rather, Selina Rathore, Aravinden Ratnakumar, Jonathan Ratoff, Deepa Rattehalli, Jason Raw, Hywel Rawlins, Gautam Ray, Adam Raymond-White, Dana Raynard, Nicola Rayner, Amy Raynsford, Salman Razvi, Zarine Razvi, Kerry Read, Sarah Read, Ajay Reddy, Anvesh Reddy, Harsha Reddy, Ravi Reddy, Aine Redfern-Walsh, Joan Redome, Anna Reed, John Reed, Andrew Rees, James Rees, Martyn Rees, Sarah Rees, Stephanie Rees, Tabitha Rees, Fiona Regan, Karen Regan, Susan Regan, Kanchan Rege, Ahmed Rehan, A Rehman, Shoib Rehman, Zainab Rehman, Ada Reid, Andrew Reid, Jennifer Reid, Jeremy Reid, Sharon Reid, Mkyla Reilly, Robert Reilly, Christina Reith, Alda Remegoso, Dinakaran Rengan, Stephen Renshaw, Remya Renu Vattekkat, Henrik Reschreiter, Mark Revels, Glynis Rewitzky, Charles Reynard, Dominic Reynish, Peter Reynolds, Piero Reynolds, Jonathan Rhodes, Naghma Riaz, Emily Rice, Matthew Rice, Mel Rich, Alison Richards, Liz Richards, Patricia Richards, Suzanne Richards, Celia Richardson, Julie Richardson, Neil Richardson, Nicky Richardson, Joanne Riches, Katie Riches, Leah Richmond, Ruth Richmond, William Ricketts, Hannah Rickman, Anna Riddell, Mohamed Ridha, Carrie Ridley, Paul Ridley, Gudrun Rieck, Linsey Rigby, Hannah Riley, Matthew Riley, Phil Riley, Zwesty V P Rimba, Dominic Rimmer, Robert Rintoul, Andrew Riordan, David Ripley, Naomi Rippon, Chloe Rishton, Michael Riste, David Ritchie, Jane Ritchie, Andy Ritchings, Pilar Rivera Ortega, Vanessa Rivers, Batool Rizvi, Syed AS Rizvi, Syed H M Rizvi, James Robb, Ian Roberts, Jane Roberts, Jean Roberts, Karen Roberts, Mark Roberts, Nicky Roberts, Philip Roberts, Rebecca Roberts, Calum Robertson, James Robertson, Jamie Robertson, Nichola Robertson, Stuart Robertson, Nicole Robin, Caroline Robinson, Emma Robinson, Gisela Robinson, Hannah Robinson, Jemima Robinson, Kate Robinson, Matthew Robinson, Ryan Robinson, Sandra Robinson, Steve Robson, Lisa Roche, Samantha Roche, Natalie Rodden, Alistair Roddick, Jack Roddy, Marion Roderick, Alison Rodger, Faye Rodger, Megan Rodger, Alicia Rodgers, Deirdre Rodgers, Natasha Rodgers, Penny Rodgers, Rocio Rodriguez-Belmonte, Nicholas Roe, Charles Roehr, Gill Rogers, Jason Rogers, Joanne Rogers, John Rogers Rogers, Leigh Rogers, Lindsay Rogers, Louise Rogers, Michaela Rogers, Paula Rogers, Susan Rogers, Thomas Rogers, Paula Rogers, Sakib Rokadiya, Lee Rollins, Jennifer Rollo, Catherine Rolls, Claire Rook, Rashmi Rook, Kevin Rooney, Lynsey Rooney, Lace P Rosaroso, Alastair Rose, Annie Rose, Steve Rose, Zoe Rose, Josephine Rosier, Jack Ross, Jenny Rossdale, Andrew Ross-Parker, Alex Rothman, Joanne Rothwell, Lindsay Roughley, Kathryn Rowan, Neil Rowan, Stephen Rowan, Anna Rowe, Louise Rowe-Leete, Benjamin Rowlands, Megan Rowley, Aparajita Roy, Subarna Roy, Anna Roynon-Reed, Sam Rozewicz, Anna Rudenko, Senthan Rudrakumar, Banu Rudran, Shannon Ruff, Prita Rughani, Sharon Rundell, Jeremy Rushmer, Darren Rusk, Peter Russell, Richard Russell, Cristina Russo, Marieke Rutgers, Aidan Ryan, Brendan Ryan, Lucy Ryan, Matthew Ryan, Pat Ryan, Phil Ryan, Declan Ryan-Wakeling, M Saad, Javeson Sabale, Suganya Sabaretnam, Noman Sadiq, Emma Sadler, Ashiq Saffy, Beth Sage, Harkiran Sagoo, Sobia Sagrir, Rajnish Saha, Sian Saha, Nikhil Sahdev, Sarvjit Sahedra, Jagdeep Sahota, Nooria Said, Sreekanth Sakthi, Hikari Sakuri, Murthy Saladi, Abdul Salam, Armorel Salberg, Erika Salciute, Gina Saleeb, Mumtaz Saleh, Hizni Salih, Laylan Salih, Sarah Salisbury, SiteEneye Saliu, Rustam Salman, Jenny Salmon, Dario Salutous, Mfon Sam, Sally Sam, Tinashe Samakomva, Renaldo Samlal, Emily Sammons, David Sammut, Mark Sammut, Zoe Sammut, Sunitha Sampath, Claire Sampson, Julia Sampson, Anda Samson, Aashna Samson, Johnson Samuel, Merna Samuel, Reena Samuel, Thomas D L Samuel, Younan Samuel, Elsward Samuels, Theo Samuels, Joanna Samways, Manjula Samyraju, Ilves Sana, Veronica Sanchez, Amada Sanchez Gonzalez, Alina Sanda-Gomez, Paul Sandajam, Peter Sandercock, Amy Sanderson, Tom Sanderson, Kuljinder Sandhu, Loveleen Sandhu, Sam Sandow, Victoria Sandrey, Sarah Sands, Mirriam Sangombe, Mathew Sanju, Filipa Santos, Rojy Santosh, Jayanta Sanyal, Aureo F Sanz-Cepero, Dinesh Saralaya, Arun Saraswatula, Joshua Sarella, Avishay Sarfatti, Rebecca Sargent, Beatrix Sari, Khatija Sarkar, Rahuldeb Sarkar, Sruthi Sarma, Zainab Sarwar, Thea Sass, Sonia Sathe, Sobitha Sathianandan, Abilash Sathyanarayanan, Lavanya S J P Sathyanarayanan, Thozhukat Sathyapalan, Prakash Satodia, Vera Saulite, Andrew Saunders, Rachel Saunders, Samantha Saunders, Anne Saunderson, Heather Savill, Karishma Savlani, Gauri Saxena, Matthew Saxton, Amrinder Sayan, Diane Scaletta, Deborah Scanlon, Jeremy Scanlon, Lyndsay Scarratt, Sean Scattergood, Alvin Schadenberg, Wendy Schneblen, Rebecca Schofield, Samuel Schofield, David Scholes, Karen Scholes, Alex Schoolmeesters, Natasha Schumacher, Nicola Schunke, Martin Schuster Bruce, Karin Schwarz, Antonia Scobie, Tim Scorrer, A Scott, Alistair Scott, Anne Scott, Catherine Scott, Christine Scott, Emily Scott, Graham Scott, Kathyn Scott, Leanne Scott, Martha Scott, Michelle Scott, Stephen Scott, Timothy Scott, Sarah Scourfield, Wendy Scrase, Angela Scullion, Therese Scullion, Emily Seager, Cathy Seagrave, Deborah Seals, Rebecca Seaman, Eleanor Sear, Isabella Seaton, Anna Seckington, Joanna Sedano, Deborah Seddon, Gabrielle Seddon, Muhammad A Seelarbokus, Christopher Sefton, Matias Segovia, Fatima Seidu, Gillian Sekadde, Faye Selby, Georgina Selby, Claire Sellar, Katharine Sellers, Joseph Selley, Victoria Sellick, Gobika Selvadurai, Brintha Selvarajah, Haresh Selvaskandan, Subothini S Selvendran, Gary Semple, Nandini Sen, Seema Sen, Aditya Sengupta, Niladri Sengupta, Susana Senra, HoJan Senya, Niranjan Setty, Abigail Seward, Teswaree Sewdin, Jack Seymour, Hussam Shabbir, Fiona Shackley, Tariq Shafi, Aashni Shah, Ahmar Shah, Anand Shah, Bhavni Shah, Momin Shah, Neil Shah, Pallav Shah, Priyank Shah, Qasim Shah, Sarfaraz H Shah, Snehal Shah, Suraj Shah, Syed Shah, Wajid Shah, Saarma Shahad, Sousan Shahi, Sipan Shahnazari, Muhammad Shahzeb, Aisha Shaibu, Zara Shaida, Amina Y Shaikh, Maliha Shaikh, Rajit Shail, Mariya Shaji, Muhammad Shakeel, Korah Shalan, Nadia Shamim, Kazi Shams, Alison Shanahan, Thomas Shanahan, Hamed Sharaf, Muhammad Sharafat, Asir Sharif, Ajay Sharma, Akhilesh Sharma, Ash Sharma, Bhawna Sharma, Mona Sharma, Ojasvi Sharma, Poonam Sharma, Rajeev Sharma, Sanjeev Sharma, Sarkhara Sharma, Shriv Sharma, Sonal Sharma, Alexander Sharp, Charles Sharp, Gemma Sharp, Paula Sharratt, Phoebe Sharratt, Katherine Sharrocks, Emma Sharrod, Christopher Shaw, Daisy Shaw, David Shaw, Deborah Shaw, Joanne Shaw, Jonathan Shaw, Lisa Shaw, Tomos G Shaw, Anna Shawcross, Jill Shawe, Lou Shayler, Sophy Shedwell, Jonathan Sheffield, Zak Shehata, Arshiya Sheik, Asif Sheikh, Noorann Sheikh, Benjamin Shelley, Sarah Shelton, Anil Shenoy, Julie Shenton, Amy Shepherd, Kate Shepherd, Lorna Shepherd, Scott Shepherd, Rhian Sheppeard, Helen Sheridan, Ray Sheridan, Samuel Sherridan, Leanne Sherris, Susanna Sherwin, Shaad Shibly, Chiaki Shioi, Anand Shirgaonkar, Kim Shirley, Adebusola Shonubi, Rob Shortman, Rohan Shotton, Sarah Shotton, Ervin Shpuza, Nora Shrestha, Karen Shuker, Jack Shurmer, Gilbert Siame, Loria Siamia, Claire Sidaway, Seshnag Siddavaram, Nasir Siddique, Sohail Siddique, Nyma Sikondari, Claudia Silva Moniz, Malcolm Sim, Theresa Simangan, Vimbai Simbi, Robert Sime, Oliver Simmons, Richard Simms, Merritt Simon, Natalie Simon, Angela Simpson, Anna Simpson, Danny Simpson, Georgina Simpson, Joanne Simpson, Kerry Simpson, Phillip Simpson, Thomas Simpson, Kathryn Simpson, Cindy Sing, Ankita Singh, Claire Singh, Jayaprakash Singh, Jyoti Singh, Lokeshwar Singh, Manjeet Singh, Nadira Singh, Pankaj Singh, Prabhsimran Singh, Salil Singh, Saurabh Singh, Parag Singhal, Bryan Singizi, Manas Sinha, Utkarsh Sinha, Guy Sisson, Sarah Sithiravel, Karthikadevi Sivakumar, Shanmugasundaram Sivakumar, Darsh Sivakumran, Sivanthi Sivanadarajah, Pasupathy-Rajah Sivasothy, Rebecca Sivers, Nicole Skehan, Robert Skelly, Orlagh Skelton, Imogen Skene, Michael Skill, Denise Skinner, Tabitha Skinner, Victoria Skinner, Agnieszka Skorko, Iwona Skorupinska, Mariola Skorupinska, Amy Slack, Katie Slack, Heather Slade, Mark Slade, Lynda Slater, Nicola Slawson, Andrew Sloan, Brendan Sloan, Derek Sloan, Geraldine Sloane, Benjamin Small, Ellen Small, Samuel Small, Karen D Smallshaw, Andy Smallwood, Carien Smit, Aileen Smith, Alex Smith, Amanda Smith, Amy Smith, Andrew Smith, Anna Smith, Camilla Smith, Catherine Smith, Chris Smith, Christopher Smith, Dominic Smith, Eleanor Smith, Harriet Smith, Hazel Smith, Helen Smith, Jacky Smith, Jessica Smith, Kate Smith, Kathryn Smith, Kelly Smith, Kerry Smith, Lara Smith, Linda Smith, Lisa Smith, Loren Smith, Maria Smith, Mel Smith, Oliver Smith, Rachel Smith, Rebecca Smith, Richard Smith, Sally Smith, Samantha Smith, Stacey Smith, Stephanie Smith, Susan Smith, Imogen Smith, John Smith, Sue Smolen, Sara Smuts, Naoise Smyth, Annette Snell, David Snell, Luke Snell, Beng So, Michelle Soan, Toluleyi Sobande, Alberto Sobrino Diaz, Basit Sohail, Bina Sohail, Herminder Sohal, Roy Soiza, Olajumoke Solademi, Krishma Solanki, Babak Soleimani, Amanda Solesbury, Reanne Solly, Louise Solomon, Subash Somalanka, Chandrashekaraiah Somashekar, Raj Sonia, Shiu-Ching Soo, Pavandeep Soor, Germanda Soothill, Jennifer Soren, Apina Sothinathan, Pragalathan Sothirajah, Najwa Soussi, Donna Southam, David Southern, Iain Southern, Louise Southern, Sara M Southin, Jessica Southwell, Thomas Southworth, Jason Sowter, Claudia Spalding, Enti Spata, Katie Spears, Mark Spears, Michelle Spence, Branwell Spencer, Gisele Spencer, Sue Spencer, Tom Spencer, Roese Spicer, Helen Spickett, Jennifer Spillane, William Spiller, Kerry Spinks, Michelle Spinks, Nick Spittle, Johanna Sporrer, Karen Spreckley, Janet Spriggs, Oliver Spring, Gemma Squires, Jack Squires, Rebecca Squires, Ram Sreenivasan, K Sri Paranthamen, Ramesh Srinivasan, Asha Srirajamadhuveeti, Vino Srirathan, Sybil Stacpoole, Louise Stadon, Jocasta Staines, Nikki Staines, Katie Stammers, Roxana Stanciu, Grazyna Stanczuk, Edward Stanton, Robyn Staples, Simon Stapley, Natalie Staplin, Adam Stark, Michelle Starr, Julie Staves, Rached Stead, Charlotte Steel, Conor Steele, John Steer, Vergnano Stefania, Paula Stefanowska, Caroline Stemp, Alison Stephens, David Stephensen, Elaine Stephenson, Monique Sterrenburg, Melanie Stevens, Will Stevens, Amy Stevenson, Andrew Stevenson, Lesley Stevenson, Sarah Stevenson, Claire Stewart, Colin Stewart, McKenna Stewart, Rachel Stewart, Richard Stewart, Jo Stickley, Gemma Stiller, Robert Stirk, Sarah Stirrup, Sarah Stock, Alexander Stockdale, Lynne Stockham, Paul Stockton, Emma Stoddard, Chris Stokes, Ben Stone, Roisin Stone, Sarah Stone, Imogen Storey, Kim Storton, Frederick Stourton, Angela Strachan, Catherine Strait, Emma Stratton, Jane Stratton, Sam Straw, Dieter Streit, Emma Stride, Sally Stringer, Sophia Strong-Sheldrake, Siske Struik, Carmel Stuart, Anna Stubbs, Harrison Stubbs, Ann Sturdy, Sharon Sturney, Matt Stuttard, Cristina Suarez, Karuna Subba, Christian P Subbe, Manjula Subramanian, Venkatram Subramanian, Chinari Subudhi, Rebecca Suckling, Srivatsan Sudershan, Gayle Sugden, Peter Sugden, Rudresh Sukla, Ali Suliman, Fatimah Suliman, Ian Sullivan, Sugrah Sultan, Jennifer Summers, Mark Summerton, Samyukta Sundar, Reka Sundhar, Edmond Sung, Nadia Sunni, Jay Suntharalingam, Amitava Sur, Dharmic Suresh, Shilpa Suresh, Michael Surtees, Crouch Susan, Danielle Suter, Helen Sutherland, Rachel Sutherland, Rebecca Sutherland, Dovile Sutinyte, Deborah Sutton, John Sutton, Sam Sutton, Mihaela Sutu, Marie-Louise Svensson, Sima Svirpliene, Andrew Swain, Thomas Swaine, Christopher Swales, Nicola Swarbrick, Tirion Swart, Stephen Sweetman, Ealish Swift, Paul Swift, Pauline Swift, Peter Swift, Rachael Swift, Rachel Swingler, Sophie Swinhoe, Katarzyna Swist-Szulik, Luke Swithenbank, Omair Syed, Catriona Sykes, Daisy Sykes, Eliot Sykes, Luke Sylvester, Dominic Symon, Andrew Syndercombe, Zoe Syrimi, Jen Syson, Gemma Szabo, Tamas Szakmany, Megan Szekely, Matthew Szeto, Maria Tadros, Amr Tageldin, Lucy Tague, Hasan Tahir, Muhammad Tahir, Zsofia Takats, Abigail Takyi, Peter Talbot, Alison Talbot -Smith, James Talbot-Ponsonby, Richard Tallent, Bradley Tallon, Adrian Tan, Bee T T Tan, Hock Tan, Huey Tan, Keith Tan, WeiTeen Tan, Anand Tana, Xiaohui Tang, Christina Tanney, Tabitha Tanqueray, Emma Tanton, Mark Taplin, Hayley Tarft, Priyal Taribagil, Obaid Tarin, Syed Tariq, David Tarpey, Lisa Tarrant, Antonia Tasiou, Elizabeth Tatam, Margaret L Tate, Kate Tatham, Vera Tavoukjian, Alexander Taylor, Beverley Taylor, Brian Taylor, Charlie Taylor, David Taylor, Elisabeth Taylor, Janet Taylor, Jennifer Taylor, Joanne Taylor, Julie Taylor, Karen Taylor, Leanne Taylor, Margaret Taylor, Matthew Taylor, Melanie Taylor, Natalie Taylor, Rachael Taylor, Rachel Taylor, Samantha Taylor, Suzanne Taylor, Suzanne Taylor, Tina Taylor, Tracey Taylor, Vicky Taylor, Michelle Taylor-Siddons, Thomas Taynton, Amelia Te, Jessica Teasdale, Julie Tebbutt, Caroline Tee, Rajni Tejwani, Adam Telfer, Vibha Teli, Jennifer Tempany, Julie Temple, Natalie Temple, Helen Tench, Yi He Teoh, Lynne Terrett, Louise Terry, Dariusz Tetla, Shirish Tewari, Daniel Tewkesbury, Joana Texeira, ChiaLing Tey, Clare Thakker, Manish Thakker, Hilary Thatcher, Andrew Thayanandan, Krishna Thazhatheyil, Eaint Thein, Lambrini Theocharidou, Phyu Thet, Kapeendran Thevarajah, Mayooran Thevendra, Nang Thiri Phoo, Yvette Thirlwall, Muthu Thirumaran, Alice Thomas, Andrew Thomas, Caradog Thomas, Emma Thomas, Enson Thomas, Esther Thomas, Helen Thomas, James Thomas, Karen Thomas, Koshy Thomas, Lucy Thomas, Rachel Thomas, Rebecca Thomas, Rhys Thomas, Ruth Thomas, Samantha Thomas, Sarah Thomas, Sherine Thomas, Tessy Thomas, Vicky Thomas, Rhian Thomas-Turner, Catherine Thompson, Christopher Thompson, Clara Thompson, Fiona Thompson, Katharine Thompson, Laura Thompson, Liz Thompson, Luke Thompson, Michael Thompson, Orla Thompson, Rebecca Thompson, Roger Thompson, Nicola Thomson, Natasha Thorn, Charlotte Thorne, Nicola Thorne, Jim Thornton, Richard Thornton, Sara Thornton, Susan Thornton, Thomas Thornton, Tracey Thornton, Christopher Thorpe, Sarah Thorpe, Paradeep Thozthumparambil, Laura Thrasyvoulou, Hannah Thraves, Elisha Thuesday, Vicky Thwaiotes, Guy Thwaites, Simon Tiberi, Jane Tidman, Serena Tieger, Carey Tierney, Caroline Tierney, Mark Tighe, Sorrell Tilbey, Amanda Tiller, John Timerick, Elizabeth Timlick, Alison Timmis, Hayley Timms, Anne-Marie Timoroksa, Samakomva Tinashe, Heather Tinkler, Marianne Tinkler, Jacqui Tipper, Helen Tivenan, Helen T-Michael, Anne Todd, Jackie Todd, Stacy Todd, Mohamed Tohfa, Melanie Tolson, Ana Luisa Tomas, Natalia Tomasova, Sharon Tomlin, Simon Tomlins, Jo Tomlinson, James Tonkin, Ivan Tonna, Catherine Toohey, Kirsty Topham, Mathew Topping, Ruhaif Tousis, Peter Tovey, Gareth Towersey, Jill Townley, Richard Tozer, Helen Tranter, Christopher Travill, Sarah Traynor, Mike Trevett, Ascanio Tridente, Sanchia Triggs, Fiona Trim, Alex Trimmings, Tom Trinick, Sven Troedson, Emily Tropman, Amy Trotter, Madeleine Trowsdale Stannard, Nigel Trudgill, Maria Truslove, Shaun Trussell, Tariq Trussell, Kyriaki Tsakiridou, Christine Tsang, Peter Tsang, Tan Tsawayo, Kyriaki K Tsilimpari, Georgios Tsinaslanidis, Simon Tso, Sally Tucker, Aisha Tufail, Redmond Tully, Grace Tunesi, Killiam Turbitt, Rezon Turel, Tolga Turgut, Claudia Turley, Alison Turnbull, Aine Turner, Ash Turner, Charlotte Turner, Gail Turner, Kate Turner, Kelly Turner, Lucy Turner, Mark Turner, Patricia Turner, Sally Turner, Samantha Turner, Susan Turner, Victoria Turner, Sharon Turney, Conor Tweed, David Tweed, Rebecca Twemlow, Emma Twohey, Bhavya Tyagi, Vedang Tyagi, Abigail Tyer, Jayne Tyler, Jennifer Tyler, Alison Tyzack, Petros Tzavaras, Mohammad S Uddin, Ruhama Uddin, Ruzena Uddin, Waqar Ul Hassan, Salamat Ullah, Sana Ullah, Sanda Ullah, Athavan Umaipalan, Judith Umeadi, Akudo Umeh, Wilfred Umeojiako, Ben Ummat, Charlotte Underwood, Jonathan Underwood, Adam Unsworth, Jasvinder Uppal, Veerpal S U Uppal, Gerry Upson, Masood Ur Rasool, Sebastian Urruela, Hiromi Uru, Miranda Usher, Rebecca Usher, Alex UsherRea, Andrew Ustianowski, Jane Uttley, Linda C Vaccari, Uddhav Vaghela, Abhay Vaidya, Abhay Vaidya, Bernardas Valecka, Jennifer Valentine, Balan Valeria, Pramodh Vallabhaneni, Luke Vamplew, Ekaterini Vamvakiti, Joannis Vamvakopoulos, Maud van de Venne, Alex van der Meer, Nora van der Stelt, Joseph Vance-Daniel, Rama Vancheeswaran, Samuel I Vandeyoon, Padma Vankayalapati, Piyush Vanmali, Chloe Vansomeren, William Van't Hoff, Sejal Vara, Stehen J Vardy, Anu Varghese, Maria Varghese, William Varney, Giulia Varnier, Valeria Vasadi, Olivia Vass, Vimal Vasu, Vasanthi Vasudevan, Manu Vatish, Heloyes Vayalaman, Christopher Vaz, Niki Veale, Sachuda Veerasamy, Bar Velan, Swati Velankar, Luxmi Velauthar, Neyme Veli, Nicola Vella, Anitha Velusamy, Ian Venables, Mavi Venditti, David Veniard, Ramya Venkataramakrishnan, Richard Venn, Robert Venn, Lyn Ventilacion, Joanne Vere, Mark Veres, Stefania Vergnano, Will Verling, Amit Verma, Rachel Vernall, Britney Vernon, Mark Vertue, Jerik Verula, Natalie Vethanayagam, Lucy Veys, Carinna Vickers, Saji Victor, Jennifer Vidler, Bavithra Vijayakumar, Vinod W Vijayaraghavan Nalini, Brigita Vilcinskaite, Neringa Vilimiene, Lynn Vinall, Sylvia Vinay, Latha Vinayakarao, Rachel Vincent, Rosie Vincent, Pritpal Virdee, Emma Virgilio, Abdullah M Virk, Elisa Visentin, Jeyakumar Visuvanathan, Karunakaran Vithian, Sorice Vittoria, Elena Vlad, Ben Vlies, Alain Vuylsteke, Eleftheria Vyras, Richard Wach, Beverley Wadams, Susan Wadd, Natalia Waddington, Kirsten Wadsworth, Syed E I Wafa, Daniel Wagstaff, Lynda Wagstaff, Dalia Wahab, Zaroug Wahbi, Abiodun Waheed Adigun, Sawan Waidyanatha, Rachel Wake, Alice Wakefield, William Wakeford, Fiona Wakinshaw, Andrew Walden, Lorna Walding, Alexandria Waldron, Gemma Walker, Harriet Walker, Ian Walker, Kevin Walker, Kim Walker, Linda Walker, Marie Walker, Olivia Walker, Rachel Walker, Rebecca Walker, Susan Walker, Rebecca Wallbutton, Jessica Wallen, Karl Wallendszus, Arabella Waller, Rosemary Waller, Gabriel Wallis, Louise Wallis, Donna Walsh, Elizabeth Walsh, Livia Walsh, Deborah Walstow, Daniel Walter, Alex Walters, Holt Walters, James Walters, Jocelyn Walters, Eileen Walton, Lucy Walton, Olivia Walton, Sharon Walton, Susan Walton, Mandy Wan, Thin Wan, Mary Wands, Rachel Wane, Frank Wang, Nick Wang, Ran Wang, Deborah Warbrick, Samantha Warburton, Deborah Ward, Emma Ward, Joanna Ward, Luke Ward, Nicola Ward, Rachael Ward, Thomas Ward, Tom Ward, Scott A Warden, Adele Wardle, Karen Wardle, Steve Wardle, Hassan Wardy, Scott Waring, Jenny Warmington, Ben Warner, Christian Warner, Lewis Warnock, Sarah Warran, Jade Warren, Lisa Warren, Yolanda Warren, Hannah Warren-Miell, Gill Warwick, Helen Wassall, Hazel J Watchorn, Holly Waterfall, Abby Waters, Donald Waters, Mark Waterstone, Catherine Watkins, Catrin Watkins, Eleanor Watkins, Karen Watkins, Lynn Watkins, Abigail Watson, Adam J R Watson, Ekaterina Watson, Eleanor Watson, Paul Watson, Rebecca Watson, Robert Watson, Malcolm Watters, Donna Watterson, Daniel Watts, John Watts, Merlin Watts, Victoria Waugh, Emma Wayman, Akhlaq Wazir, Mark Weatherhead, Nick Weatherly, Hayley Webb, Kathryn Webb, Kylie Webb, Stephen Webb, Cheryl Websdale, Deborah Webster, Ian Webster, Tim Webster, Ling Wee, Rebecca Weerakoon, Thanuja Weerasinghe, Janaka Weeratunga, Maria Weetman, Shuying Wei, Immo Weichert, Hugh Welch, James Welch, Leanne Welch, Steven Welch, Samantha Weller, Lucy Wellings, Brian Wells, Susan Wellstead, Berni Welsh, RIchard Welsh, Ingeborg Welters, Rachael Welton, Lauren Wentworth, Kate Wesseldine, Jim Wesson, Magdelena West, Raha West, Ruth West, Sophie West, Luke Western, Ruth Westhead, Heather Weston, Alice Westwood, Bill Wetherill, Sharon Wheaver, Helen Wheeler, Ben Whelan, Matthew Whelband, Amanda Whileman, Alison Whitcher, Andrew White, Benjamin White, Christopher White, Duncan White, Emily White, James White, Jonathan White, Katie White, Marie White, Nick White, Sarah White, Sonia White, Tracey White, Catherine Whitehead, Anne Whitehouse, Claire Whitehouse, Tony Whitehouse, Julia Whiteley, Sophie Whiteley, Gabriel Whitlingum, David Whitmore, Elizabeth Whittaker, Lindsay Whittam, Andrew Whittingham-Hirst, Ashley Whittington, Helen Whittle, Robert Whittle, Suzanne Whyte, Eunice Wiafe, Lou Wiblin, John Widdrington, Jason Wieboldt, Hannah Wieringa, Cornelia Wiesender, Laura Wiffen, Laura Wiffen, Andrew Wight, Christopher Wignall, Danielle Wilcock, Emma Wilcock, Louise Wilcox, Laura Wild, Stephen Wild, Michael Wilde, Peter Wilding, Tracey Wildsmith, Joe Wileman, Donna Wiles, Joy Wiles, Kate Wiles, Elva Wilhelmsen, Thomas Wiliams, Janet Wilkie, David Wilkin, Hannah Wilkins, Joy Wilkins, Suzanne Wilkins, Iain Wilkinson, Lesley Wilkinson, Nicola Wilkinson, Sophia Wilkinson, Susan Wilkinson, Tim Wilkinson, Sylvia Willetts, Aimee Williams, Alexandra Williams, Alison Williams, Angharad Williams, Ava Williams, Carl Williams, Caroline V Williams, Claire Williams, Dewi Williams, Gail Williams, Gemma Williams, Gina Williams, Hannah Williams, James Williams, Jennie Williams, John Williams, Joseph Williams, Karen Williams, Kathryn Williams, Marie Williams, Matthew Williams, Patricia Williams, Penny Williams, Rachael Williams, Rupert Williams, Samson Williams, Sarah Williams, Sophie Williams, Tamanna Williams, Annie Williamson, Catherine Williamson, Dawn Williamson, James D Williamson, Rachel Williamson, Helen Williamson, Elizabeth Willis, Emily Willis, Heather Willis, Herika Willis, Herika Willis, Joanna Willis, Louise Wills, Lucy Willsher, Catherine Willshire, Francesca Willson, Alison Wilson, Andrea Wilson, Antoinette Wilson, Billy Wilson, Eve Wilson, James Wilson, Karen Wilson, Kate Wilson, Lucinda Wilson, Mark Wilson, Toni Wilson, Marlar Win, Tin T Win, Wut Y W Win, Lucinda Winckworth, Laura Winder, Piers Winder, Kerry Winham-Whyte, Helen Winmill, Simon Winn, Carmen Winpenny, Helen Winslow, Helen Winter, Jonathan Winter, Barbara Winter-Goodwin, Stephen Wisdom, Matthew Wise, Martin Wiselka, Rebecca Wiseman, Sophie Wiseman, Steven Wishart, Holly Wissett, Eric Witele, Nicholas Withers, Janet Wittes, Donna Wixted, Therese Wodehouse, Will Wolf, Nicola Wolff, Kirsten Wolffsohn, Rebecca Wolf-Roberts, Elena Wolodimeroff, Adam Wolstencroft, Alan Wong, Charlotte Wong, Chi-Hung Wong, Edwin Wong, Jessica S Y Wong, Kit Y Wong, Mei Yin Wong, Nick Wong, Sam Wong, Amanda Wood, Caroline Wood, Dianne Wood, Fiona Wood, Hannah Wood, Jennifer Wood, Joe Wood, Kathryn Wood, Lisa Wood, Louise Wood, Michelle Wood, Stephen Wood, Tracy Wood, Rebecca Woodfield, Christopher Woodford, Elizabeth Woodford, Jill Woodford, Louise Woodhead, Timothy Woodhead, Philip Woodland, Marc Woodman, Jane Woods, Katherine Woods, Sarah Woods, Zoe Woodward, Megan Woolcock, Gemma Wooldridge, Rebecca Woolf, Chris Woollard, Christopher Woollard, Louisa Woollen, Emma Woolley, Jade Woolley, Katharine Wooodall, Daniel Woosey, Dan Wootton, Joanne Wootton, Daniel Worley, Stephy Worton, Jonathan Wraight, Maria Wray, Tim Wreford-Bush, Joanne Wren, Kim Wren, Lynn Wren, Caroline Wrey Brown, Catherine Wright, Demi Wright, Francesca Wright, Imogen Wright, Lianne Wright, Rachel Wright, Rebecca Wright, Stephanie Wright, Tim Wright, Caroline Wroe, Hannah Wroe, Henry Wu, Peishan Wu, Pensee Wu, Jonathan Wubetu, Retno Wulandari, Craig Wyatt, Frederick Wyn-Griffiths, Inez Wynter, Bindhu Xavier, Arnold Xhikola, Zhongyang Xia, Masseh Yakubi, May Yan, Freda Yang, Yingjia Yang, Michael Yanney, Woei Lin Yap, Nabil Yaqoob, Salima Yasmin, Bryan Yates, David Yates, Edward Yates, Helen Yates, Julie Yates, Mark Yates, Charlotte Yearwood Martin, Khin Yein, Fiona Yelnoorkar, Peter Yew, Kawai Yip, Laura Ylquimiche, Laura Ylquimiche Melly, Inez Ynter, H Yong, Jemma Yorke, Jasmine Youens, Abdel Younes Ibrahim, Eoin Young, Gail Young, Louise Young, Asfand Yousafzar, Sajeda Youssouf, Ahmed Yousuf, Chrissie Yu, Bernard Yung, Daniel Yusef, Said Yusef, Intekhab Yusuf, Anna-Sophia Zafar, Silvia Zagalo, Su Zaher, Aqsa Zahoor, Kareem Zaki, Nabhan Zakir, Kasia Zalewska, Ane Zamalloa, Mohsin Zaman, Raisa Zaman, Shakir Zaman, Julie Zamikula, Louise Zammit, Marie Zammit-Mangion, Esther Zebracki, Daniel Zehnder, Lisa Zeidan, Xiaobei Zhao, Dongling Zheng, Doreen Zhu, Madiha Zia, Omar Zibdeh, Rabia Zill-E-Huma, Ei Thankt Zin, Eleanor Zinkin, Vivian Zinyemba, Christos Zipitis, Arkadiusz Zmierczak, Azam Zubir, Naz Zuhra, Rasha Zulaikha, Sabrina Zulfikar, Carol Zullo, Ana Zuriaga-Alvaro

## Abstract

**Background:**

In this study, we aimed to evaluate the effects of tocilizumab in adult patients admitted to hospital with COVID-19 with both hypoxia and systemic inflammation.

**Methods:**

This randomised, controlled, open-label, platform trial (Randomised Evaluation of COVID-19 Therapy [RECOVERY]), is assessing several possible treatments in patients hospitalised with COVID-19 in the UK. Those trial participants with hypoxia (oxygen saturation <92% on air or requiring oxygen therapy) and evidence of systemic inflammation (C-reactive protein ≥75 mg/L) were eligible for random assignment in a 1:1 ratio to usual standard of care alone versus usual standard of care plus tocilizumab at a dose of 400 mg–800 mg (depending on weight) given intravenously. A second dose could be given 12–24 h later if the patient's condition had not improved. The primary outcome was 28-day mortality, assessed in the intention-to-treat population. The trial is registered with ISRCTN (50189673) and ClinicalTrials.gov (NCT04381936).

**Findings:**

Between April 23, 2020, and Jan 24, 2021, 4116 adults of 21 550 patients enrolled into the RECOVERY trial were included in the assessment of tocilizumab, including 3385 (82%) patients receiving systemic corticosteroids. Overall, 621 (31%) of the 2022 patients allocated tocilizumab and 729 (35%) of the 2094 patients allocated to usual care died within 28 days (rate ratio 0·85; 95% CI 0·76–0·94; p=0·0028). Consistent results were seen in all prespecified subgroups of patients, including those receiving systemic corticosteroids. Patients allocated to tocilizumab were more likely to be discharged from hospital within 28 days (57% *vs* 50%; rate ratio 1·22; 1·12–1·33; p<0·0001). Among those not receiving invasive mechanical ventilation at baseline, patients allocated tocilizumab were less likely to reach the composite endpoint of invasive mechanical ventilation or death (35% *vs* 42%; risk ratio 0·84; 95% CI 0·77–0·92; p<0·0001).

**Interpretation:**

In hospitalised COVID-19 patients with hypoxia and systemic inflammation, tocilizumab improved survival and other clinical outcomes. These benefits were seen regardless of the amount of respiratory support and were additional to the benefits of systemic corticosteroids.

**Funding:**

UK Research and Innovation (Medical Research Council) and National Institute of Health Research.

## Introduction

The majority of SARS-CoV-2 infections are either asymptomatic or result in only mild disease.^1^ However, a substantial proportion of infected individuals develop a respiratory illness requiring hospital care, which can progress to critical illness with hypoxic respiratory failure requiring prolonged ventilatory support. Among COVID-19 patients admitted to UK hospitals in spring, 2020, the case fatality rate was over 26%, and was in excess of 37% in patients requiring invasive mechanical ventilation.^2^

Hypoxic respiratory failure in patients with COVID-19 is associated with evidence of systemic inflammation, including release of pro-inflammatory cytokines, such as interleukin (IL)-1, IL-6, and tumour necrosis factor α, and elevated concentrations of D-dimer, ferritin, and C-reactive protein (CRP).^3^, ^4^ The host immune response is thought to play a key role in driving an acute inflammatory pneumonic process with diffuse alveolar damage, myeloid cell infiltrates, and microvascular thrombosis.^5^ The beneficial effects of dexamethasone and other corticosteroids in COVID-19 patients with hypoxic lung damage suggest that other, more specific, immunomodulatory agents might provide additional improvements in clinical outcomes.^6^, ^7^

Tocilizumab is a recombinant humanised anti-IL-6 receptor monoclonal antibody that inhibits the binding of IL-6 to both membrane and soluble IL-6 receptors, blocking IL-6 signalling and reducing inflammation. Tocilizumab is licensed in the UK as an intravenous treatment for patients with rheumatoid arthritis and for people with chimeric antigen receptor T-cell-induced severe or life-threatening cytokine release syndrome. Randomised trials of tocilizumab in COVID-19 have so far shown mixed results for 28-day mortality: seven small trials reported no benefit and the somewhat larger REMAP-CAP trial reported a benefit in patients requiring organ support.^8^, ^9^, ^10^, ^11^, ^12^, ^13^, ^14^, ^15^ Here we report the results of a large randomised, controlled trial aimed at evaluating the effects of tocilizumab in adult patients hospitalised with severe COVID-19 characterised by hypoxia and substantial inflammation.

**Research in context**

**Evidence before this study**

We searched MEDLINE, Embase, and MedRxiv from inception up to March 5, 2021, for clinical trials or meta-analyses evaluating the effect of interleukin-6 inhibitor treatment on patients with COVID-19 using the search terms (“COVID-19” OR “COVID” OR “SARS-CoV-2” OR “2019-nCoV” OR “coronavirus”) AND (“tocilizumab” OR “sarilumab” OR “interleukin-6 inhibitor” or “IL-6 inhibitor”).

We identified eight relevant randomised trials that compared tocilizumab with usual care or placebo in hospitalised patients with COVID-19. All were assessed as at low risk of bias. Of these trials, only the REMAP-CAP trial in critically ill patients found a significant reduction in 28-day mortality with tocilizumab. A meta-analysis of these eight trials, which included a total of 439 deaths among 2379 patients showed no significant difference in 28-day mortality (death rate ratio 0·89, 95% CI 0·72–1·11).

**Added value of this study**

The Randomised Evaluation of COVID-19 Therapy (RECOVERY) trial is the largest randomised trial of the effect of tocilizumab in hospitalised patients with COVID-19. We found that in 4116 COVID-19 patients with hypoxia and a raised C-reactive protein, tocilizumab reduced 28-day mortality, increased the probability of discharge within 28 days, and, among patients who were not receiving invasive mechanical ventilation at randomisation, reduced the probability of progression to the composite outcome of invasive mechanical ventilation or death. The benefits were in addition to corticosteroids and consistent in all subgroups, regardless of the amount of respiratory support.

**Implications of all the available evidence**

Our finding shows that tocilizumab improves survival and other clinical outcomes in a broad group of patients hospitalised with COVID-19 and that these benefits are additional to those of corticosteroids.

## Methods

### Study design and participants

The Randomised Evaluation of COVID-19 Therapy (RECOVERY) trial is an investigator-initiated, individually randomised, controlled, open-label, platform trial to evaluate the effects of potential treatments in patients hospitalised with COVID-19. Details of the trial design and results for other possible treatments have been published previously.^6^, ^16^, ^17^, ^18^ The trial is being done in acute National Health Service hospitals in the UK, supported by the National Institute for Health Research Clinical Research Network (appendix pp 2–25). The trial is coordinated by the Nuffield Department of Population Health at University of Oxford (Oxford, UK), the trial sponsor. The trial is being done in accordance with the principles of the International Conference on Harmonisation–Good Clinical Practice guidelines and is approved by the UK Medicines and Healthcare products Regulatory Agency and the Cambridge East Research Ethics Committee. The protocol, statistical analysis plan, and additional information are available on the study website. This report is limited to adult patients. The randomised assessment of tocilizumab in children younger than 18 years is ongoing.

Patients admitted to hospital were eligible for the study if they had clinically suspected or laboratory confirmed SARS-CoV-2 infection and no medical history that might, in the opinion of the attending clinician, put the patient at substantial risk if they were to participate in the trial. Written informed consent was obtained from all patients, or their legal representative if they were too unwell or unable to provide consent.

### Randomisation and masking

Data were collected at study entry using a web-based case report form that included demographics and major comorbidities (appendix p 32). All eligible and consenting patients received usual standard of care and underwent an initial (main) randomisation comprising up to three parts in a factorial design (appendix pp 29–30): part 1, no additional treatment versus either dexamethasone, lopinavir–ritonavir, hydroxychloroquine, azithromycin, or colchicine; part 2, no additional treatment versus either convalescent plasma or REGN-COV2 (a combination of two monoclonal antibodies directed against SARS-CoV-2 spike protein); and part 3, no additional treatment versus aspirin. Over time, treatment groups were added to and removed from the protocol (appendix pp 26–29), and not all treatments were available at every hospital. Similarly, not all treatments were suitable for some patients (eg, owing to comorbid conditions or concomitant medication). In any of these cases, randomisation was between fewer groups.

Up to 21 days after the main randomisation and regardless of treatment allocation, RECOVERY trial participants with clinical evidence of progressive COVID-19 (defined as oxygen saturation <92% on room air or receiving oxygen therapy, and CRP ≥75 mg/L) could be considered for randomisation to tocilizumab versus usual care alone. Baseline data collected for this randomisation included amount of respiratory support, markers of progressive COVID-19 (including most recent oxygen saturation, CRP, ferritin, and creatinine result before second randomisation), suitability for the study treatment, and treatment availability at the site (appendix pp 33–34). For some patients, tocilizumab was unavailable at the hospital at the time of enrolment or was considered by the managing physician to be either definitely indicated or definitely contraindicated. In such cases, the patients were not eligible for the tocilizumab randomisation. Patients with known hypersensitivity to tocilizumab, evidence of active tuberculosis infection or clear evidence of active bacterial, fungal, viral, or other infection (besides COVID-19) were not eligible for randomisation to tocilizumab.

Patients who were eligible for randomisation to tocilizumab were assigned to either usual standard of care or usual standard of care plus tocilizumab in a 1:1 ratio by means of web-based simple (unstratified) randomisation with allocation concealed until after randomisation. Allocated treatment was prescribed by the managing doctor. Roche Products (Welwyn Garden City, UK) supported the trial through provision of tocilizumab. Participants and local study staff were not masked to the allocated treatment. The steering committee, investigators, and all others involved in the trial were masked to the outcome data during the trial.

### Procedures

Patients allocated to tocilizumab were to receive tocilizumab as a single intravenous infusion over 60 min. The dose of tocilizumab was established by bodyweight (800 mg if weight >90 kg; 600 mg if weight >65 and ≤90 kg; 400 mg if weight >40 and ≤65 kg; and 8 mg/kg if weight ≤40 kg). A second dose could be given 12–24 h later if, in the opinion of the attending clinician, the patient's condition had not improved.

A single online follow-up form was completed when participants were discharged, had died, or at 28 days after the initial randomisation, whichever occurred earliest (appendix pp 35–41). Information was recorded on adherence to allocated study treatment, receipt of other COVID-19 treatments, duration of admission, receipt of respiratory or renal support, and vital status (including cause of death). In addition, routine health-care and registry data were obtained for the full follow-up period, including information on vital status (with date and cause of death), discharge from hospital, receipt of respiratory support, or renal replacement therapy.

### Outcomes

Outcomes were assessed at 28 days after randomisation to tocilizumab versus usual care alone, with further analyses specified at 6 months. The primary outcome was all-cause mortality. Secondary outcomes were time to discharge from hospital, and, among patients not receiving invasive mechanical ventilation at randomisation, receipt of invasive mechanical ventilation (including extracorporeal membrane oxygenation) or death. Prespecified subsidiary clinical outcomes were use of non-invasive respiratory support (defined as high-flow nasal oxygen, continuous positive airway pressure, or non-invasive ventilation), time to successful cessation of invasive mechanical ventilation (defined as cessation of invasive mechanical ventilation within, and survival to, 28 days), and use of renal dialysis or haemofiltration. Prespecified safety outcomes included cause-specific mortality and major cardiac arrhythmia. Information on suspected serious adverse reactions was collected in an expedited fashion to comply with regulatory requirements.

### Statistical analysis

In accordance with the statistical analysis plan (version 2.1, appendix pp 93–117), an intention-to-treat comparison was done between patients who entered the randomised comparison of tocilizumab versus usual care. For the primary outcome of 28-day mortality, the log-rank observed minus expected statistic and its variance were used to test the null hypothesis of equal survival curves (ie, the log-rank test) and to calculate the one-step estimate of the average mortality rate ratio. We constructed Kaplan-Meier survival curves to display cumulative mortality over the 28-day period. We used the same method to analyse time to hospital discharge and successful cessation of invasive mechanical ventilation, with patients who died in hospital right-censored on day 29. For the prespecified composite secondary outcome of invasive mechanical ventilation or death within 28 days (among those not receiving invasive mechanical ventilation at randomisation) and the subsidiary clinical outcomes of receipt of ventilation and receipt of haemodialysis or haemofiltration, the precise dates were not available and so the risk ratio was estimated instead.

Prespecified analyses of the primary outcome were done in subgroups defined by six characteristics at the time of randomisation: age, sex, ethnicity, amount of respiratory support, days since symptom onset, and use of systemic corticosteroids (including dexamethasone). Observed effects within subgroup categories were compared by means of a χ^2^ test for heterogeneity or trend, in accordance with the prespecified analysis plan.

Estimates of rate and risk ratios are shown with 95% CIs. All p values are two-sided and are shown without adjustment for multiple testing. The full database is held by the study team which collected the data from study sites and did the analyses at the Nuffield Department of Population Health, University of Oxford (Oxford, UK).

Before commencement of the randomisation to tocilizumab versus usual care, the trial steering committee determined that if 28-day mortality in the usual care group was above 25% then recruitment of around 4000 patients to this comparison would provide 90% power at two-sided p=0·01 to detect a proportional reduction in 28-day mortality of one-fifth. Consequently, Roche Products provided sufficient treatment for 2000 patients to receive tocilizumab. The trial steering committee, masked to the results, closed recruitment to the tocilizumab comparison at the end of Jan 24, 2021, as over 4000 patients had been randomly assigned.

For the primary outcome of 28-day mortality, the results from RECOVERY were subsequently included in a meta-analysis of results from all previous randomised trials of tocilizumb versus usual care in patients with COVID-19. For each trial, we compared the observed number of deaths among patients allocated tocilizumab with the expected number if all patients were at equal risk (ie, we calculated the observed minus expected statistic [o–e], and its variance v). For RECOVERY, these were taken as the log-rank observed minus expected statistic and its variance but for other trials, where the exact timing of each death was not available, these were calculated from standard formulae for 2 × 2 contingency tables. We then combined trial results using the log of the mortality rate ratio calculated as the inverse-variance weighted average S/V with variance 1/V (and hence with 95% CI S/V ±1·96/√V), where S is the sum over all trials of (O–E) and V is the sum over all trials of v.^19^ Analyses were done by means of SAS version 9.4 and R version 3.4. The trial is registered with ISRCTN (50189673) and ClinicalTrials.gov (NCT04381936).

### Role of the funding source

Neither the funders of the study nor Roche Products had any role in study design, data collection, data analysis, data interpretation, or writing of the report. Roche Products supported the study through the supply of tocilizumab and reviewed the draft publication for factual accuracy relating to tocilizumab.

## Results

Between April 23, 2020, and Jan 24, 2021, 4116 (19%) of 21 550 patients enrolled into the RECOVERY trial at one of the 131 sites in the UK participating in the tocilizumab comparison were eligible for random assignment. 2022 patients were randomly allocated to tocilizumab and 2094 were randomly allocated to usual care. The mean age of these participants was 63·6 years (SD 13·6). At randomisation, 562 (14%) of 4116 patients were receiving invasive mechanical ventilation, 1686 (41%) of 4116 were receiving non-invasive respiratory support (including high-flow nasal oxygen, continuous positive airway pressure, and non-invasive ventilation), and 1868 (45%) of 4116 were receiving no respiratory support other than simple oxygen therapy (nine of these patients were reportedly not receiving oxygen at randomisation; table 1). Median CRP was 143 (IQR 107–204) mg/L. 82% of patients were reported to be receiving corticosteroids at randomisation (and 97% of the patients enrolled since the announcement of the dexamethasone result from RECOVERY in June, 2020).

**Table 1:** Baseline characteristics

		**Tocilizumab group (n=2022)**	**Usual care group (n=2094)**
Age, years		63·3 (13·7)	63·9 (13·6)
	≥18 to <70	1331 (66%)	1355 (65%)
	≥70 to <80	478 (24%)	480 (23%)
	≥80	213 (11%)	259 (12%)
Sex			
	Male	1337 (66%)	1437 (69%)
	Female*	685 (34%)	657 (31%)
Ethnicity			
	White	1530 (76%)	1597 (76%)
	Black, Asian, or minority ethnic	354 (18%)	378 (18%)
	Unknown	138 (7%)	119 (6%)
Number of days since symptom onset		9 (7–13)	10 (7–14)
Number of days since hospitalisation		2 (1–5)	2 (1–5)
Oxygen saturation		94% (92–96)	94% (91–95)
Respiratory support at second randomisation			
	No ventilator support†	935 (46%)	933 (45%)
	Non-invasive ventilation‡	819 (41%)	867 (41%)
	Invasive mechanical ventilation§	268 (13%)	294 (14%)
Biochemistry at second randomisation			
	Latest C-reactive protein, mg/L	143 (107–203)	144 (106–205)
	Ferritin, ng/mL	947 (497–1599)	944 (507–1533)
	Creatinine, μmol/L	77 (62–98)	77 (62–100)
Previous diseases			
	Diabetes	569 (28%)	600 (29%)
	Heart disease	435 (22%)	497 (24%)
	Chronic lung disease	473 (23%)	484 (23%)
	Tuberculosis	3 (<1%)	5 (<1%)
	HIV	7 (<1%)	8 (<1%)
	Severe liver disease¶	14 (1%)	10 (<1%)
	Severe kidney impairment||	118 (6%)	99 (5%)
	Any of the above	1100 (54%)	1163 (56%)
SARS-CoV-2 test result			
	Positive	1922 (95%)	2005 (96%)
	Negative	69 (3%)	71 (3%)
	Not known	31 (2%)	18 (1%)
First randomisation**			
	Number of days since first randomisation	0 (0–1)	0 (0–1)
Part A allocation			
	Usual care	839 (41%)	869 (41%)
	Lopinavir–ritonavir	51 (3%)	64 (3%)
	Dexamethasone	49 (2%)	45 (2%)
	Hydroxychloroquine	37 (2%)	38 (2%)
	Azithromycin	197 (10%)	177 (8%)
Use of systemic corticosteroids††			
	Yes	1664 (82%)	1721 (82%)
	No	357 (18%)	367 (18%)
	Unknown	1 (<1%)	6 (<1%)

**Da**ta are mean (SD), n (%), or median (IQR). Information on sex, ethnicity, and SARS-CoV-2 test result were recorded on the main randomisation form when patients first entered the study. All other information was recorded on the second randomisation form (when patients were randomly assigned to tocilizumab *vs* usual care alone).

* Includes ten pregnant women.

† Includes nine patients not receiving any oxygen and 1859 patients receiving low-flow oxygen.

‡ Includes patients receiving high-flow nasal oxygen, continuous positive airway pressure, or other non-invasive ventilation.

§ Includes patients receiving invasive mechanical ventilation or extracorporeal membranous oxygenation.

¶ Defined as requiring ongoing specialist care.

|| Defined as estimated glomerular filtration rate <30 mL/min per 1·73 m^2^.

** 2631 participants were randomly assigned into part B and 1615 into part C of the first randomisation.

†† Information on use of corticosteroids was collected from June 18, 2020, onwards following announcement of the results of the dexamethasone comparison from the RECOVERY trial. Participants undergoing first randomisation before this date (and who were not allocated to dexamethasone) are assumed not to be receiving systemic corticosteroids.

**Figure 1:**
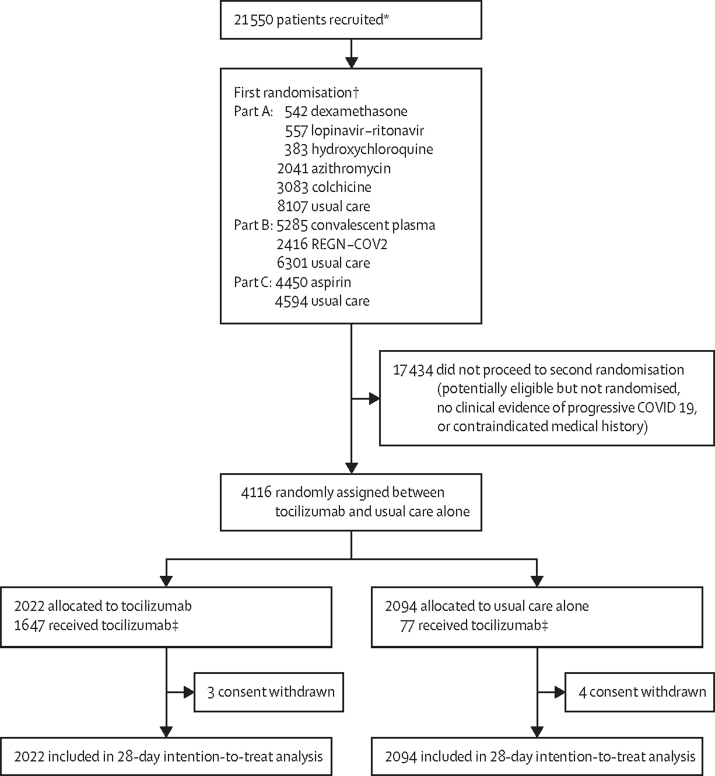
Trial profile REGN-COV2=a combination of two monoclonal antibodies directed against SARS-CoV-2 spike protein. *Number of adult patients recruited at a site activated for the tocilizumab comparison. †The first randomisation comprised up to three factorial elements such that an eligible patient could be entered into between one and three randomised comparisons, depending on the then current protocol, the patient's suitability for particular treatments, and the availability of the treatment at the site. Median time between first and second randomisation was 0·3 h (IQR 0·1−25·3). ‡1964 (97%) of 2022 patients of those allocated to tocilizumab and 2049 (98%) of 2094 of those allocated to usual care had a completed follow-up form at time of analysis.

The follow-up form was completed for 1964 (97%) of 2022 randomly assigned patients in the tocilizumab group and 2049 (98%) of 2094 patients in the usual care group. Among patients with a completed follow-up form, 1647 (84%) of 1964 allocated to tocilizumab and 77 (4%) of 2049 of those allocated to usual care received at least one dose of tocilizumab (or sarilumab, another IL-6 antagonist; figure 1; appendix p 44). 565 (29%) of 1964 patients in the tocilizumab group and 17 (1%) of 2049 in the usual care group received more than one dose of tocilizumab (or sarilumab). Use of other treatments for COVID-19 during the 28 days after randomisation was similar among patients allocated tocilizumab and among those allocated usual care (appendix p 44). Follow-up for the primary and secondary outcomes was complete for 99% of randomised participants.

**Figure 2:**
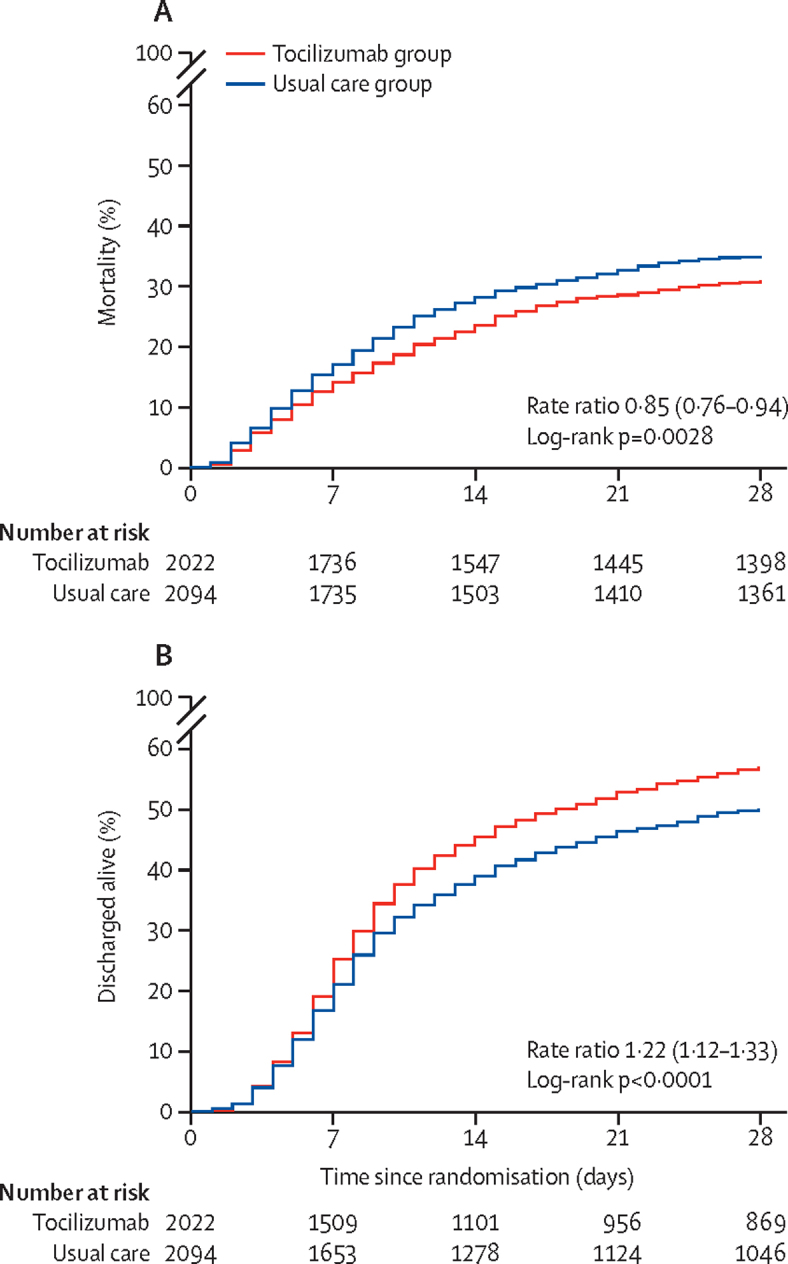
Effect of allocation to tocilizumab on 28-day mortality (A) and discharge from hospital within 28 days of randomisation (B)

Allocation to tocilizumab was associated with a significant reduction in the primary outcome of 28-day mortality compared with usual care alone (621 [31%] of 2022 patients in the tocilizumab group *vs* 729 (35%) of 2094 patients in the usual care group; rate ratio 0·85; 95% CI, 0·76–0·94; p=0·0028; figure 2A). In an exploratory analysis restricted to the 3927 (95%) patients with a positive SARS-CoV-2 test result, the result was similar (rate ratio 0·86, 95% CI 0·77–0·97; p=0·0098).

Allocation to tocilizumab was associated with a greater probability of discharge from hospital within 28 days (57% *vs* 50%; rate ratio 1·22, 1·12–1·33, p<0·0001; figure 2B and table 2). Among those not on invasive mechanical ventilation at baseline, allocation to tocilizumab was associated with a reduction in the risk of progressing to the prespecified composite secondary outcome of invasive mechanical ventilation or death when compared with usual care alone (35% *vs* 42%, risk ratio 0·84, 0·77–0·92, p<0·0001; table 2).

**Table 2:** Effect of allocation to tocilizumab on main study outcomes

		**Treatment allocation**		**RR (95% CI)**	**p value**
		Tocilizumab group (n=2022)	Usual care group (n=2094)		
**Primary outcome**					
28-day mortality		621 (31%)	729 (35%)	0·85 (0·76–0·94)	0·0028
**Secondary outcomes**					
Median time to being discharged, days		19	>28	··	··
Discharged from hospital within 28 days		1150 (57%)	1044 (50%)	1·22 (1·12–1·33)	<0·0001
Receipt of invasive mechanical ventilation or death*		619/1754 (35%)	754/1800 (42%)	0·84 (0·77–0·92)	<0·0001
	Invasive mechanical ventilation	265/1754 (15%)	343/1800 (19%)	0·79 (0·69–0·92)	0·0019
	Death	490/1754 (28%)	580/1800 (32%)	0·87 (0·78–0·96)	0·0055
**Subsidiary clinical outcomes**					
Receipt of ventilation†		290/935 (31%)	323/933 (35%)	0·90 (0·79–1·02)	0·10
	Non-invasive ventilation	281/935 (30%)	309/933 (33%)	0·91 (0·79–1·04)	0·15
	Invasive mechanical ventilation	67/935 (7%)	86/933 (9%)	0·78 (0·57–1·06)	0·11
Successful cessation of invasive mechanical ventilation‡		95/268 (35%)	98/294 (33%)	1·08 (0·81–1·43)	0·60
Use of haemodialysis or haemofiltration§		120/1994 (6%)	172/2065 (8%)	0·72 (0·58–0·90)	0·0046

Data are n (%), n/N (%), or median (IQR) unless stated otherwise. RR=rate ratio for the outcomes of 28-day mortality, hospital discharge, and successful cessation of invasive mechanical ventilation, and risk ratio for other outcomes.

* Analyses include only those on no ventilator support or non-invasive ventilation at second randomisation.

† Analyses include only those on no ventilator support at second randomisation.

‡ Analyses restricted to those on invasive mechanical ventilation at second randomisation.

§ Analyses exclude those on haemodialysis or haemofiltration at second randomisation.

**Figure 3:**
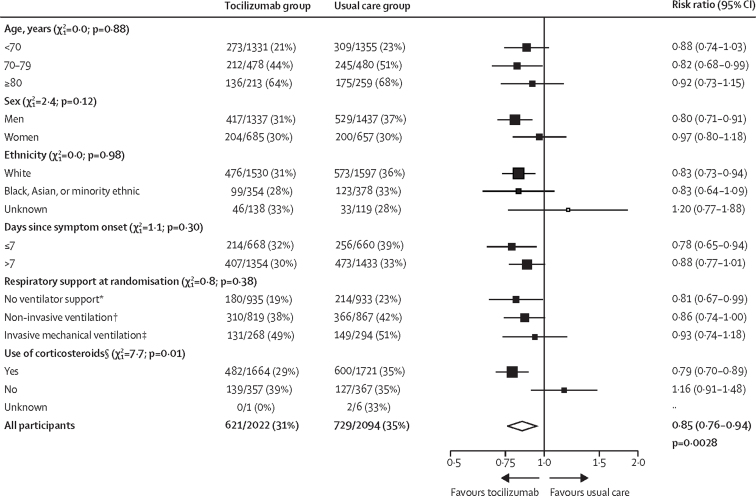
Effect of allocation to tocilizumab on 28-day mortality by baseline characteristics Subgroup-specific rate ratio estimates are represented by squares (with areas of the squares proportional to the amount of statistical information) and the lines through them correspond to the 95% CIs. *Includes nine patients not receiving any oxygen and 1859 patients receiving simple oxygen only. †Includes patients receiving high-flow nasal oxygen, continuous positive airway pressure ventilation, and other non-invasive ventilation. ‡Includes patients receiving invasive mechanical ventilation and extracorporeal membranous oxygenation. §Information on use of corticosteroids was collected from June 18, 2020, onwards following announcement of the results of the dexamethasone comparison from the RECOVERY trial. Participants undergoing first randomisation before this date (and who were not allocated to dexamethasone) are assumed not to be receiving systemic corticosteroids. In a model adjusted for all six baseline subgroups (in the categories shown) the overall rate ratio was 0·88 (95% CI 0·79−0·98).

**Figure 4:**
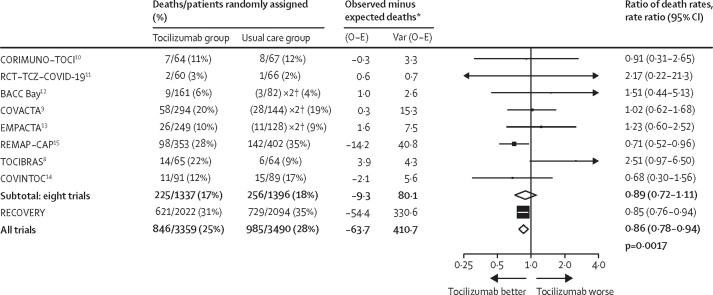
Meta-analysis of mortality in randomised, controlled trials of tocilizumab in patients hospitalised with COVID-19 O–E=observed–expected. Var=variance. *Log–rank O–E for RECOVERY, O–E from 2 × 2 contingency tables for the other trials. Rate ratio is calculated by taking ln rate ratio to be (O–E)/V with normal variance 1/V, where V=Var (O–E). Subtotals or totals of (O–E) and of V yield inverse-variance weighted averages of the ln rate ratio values. †For balance, controls in the 2:1 studies count twice in the control totals and subtotals, but do not count twice when calculating their O–E or V values. Heterogeneity between RECOVERY and eight previous trials combined, χ_1_^2^=0·2 (p=0·7).

We observed similar results across all prespecified subgroups (figure 3, appendix pp 48–49), including the amount of respiratory support at randomisation (figure 3). Given the number of hypothesis tests done, the suggestion of a larger proportional mortality reduction among those receiving a corticosteroid compared with those not (interaction p=0·01) might reflect the play of chance. An exploratory analysis showed that the effects of tocilizumab on 28-day mortality were similar for those randomly assigned ≤2 or >2 days since hospitalisation (interaction p=0·89). In eight previous trials of tocilizumab versus usual care, which included a total of 439 deaths among 2379 patients, allocation to tocilizumab was associated with a non-significant 11% reduction in mortality (rate ratio 0·89, 0·72–1·11; figure 4). After inclusion of the 28-day mortality results from RECOVERY into this meta-analysis, the mortality rate ratio from the nine trials was 0·86 (0·78–0·94), p=0·0017.

In prespecified subsidiary analyses, we found no significant effect of tocilizumab on subsequent receipt of non-invasive respiratory support or invasive mechanical ventilation among those on no respiratory support at randomisation (table 2, appendix p 50). Nor was there a significant effect on the rate of successful cessation of invasive mechanical ventilation among those on invasive mechanical ventilation at randomisation. However, allocation to tocilizumab reduced the use of haemodialysis or haemofiltration (6% vs 8%, risk ratio 0·72, 0·58–0·90, p=0·0046; table 2) among those not receiving haemodialysis or haemofiltration at randomisation. There was no evidence of excess deaths from non-COVID infections or other causes (appendix p 45). We observed no significant differences in the frequency of new cardiac arrhythmias (appendix p 46). There were three reports of serious adverse reactions believed to be related to tocilizumab: one each of otitis externa, *Staphylococcus aureus* bacteraemia, and lung abscess, all of which resolved with standard treatment.

## Discussion

The results of this large, randomised trial indicate that tocilizumab is an effective treatment for hospitalised COVID-19 patients who have hypoxia and evidence of inflammation (CRP ≥75 mg/L). Treatment with tocilizumab improved survival and the chances of discharge from hospital by 28 days, and reduced the chances of progressing to require invasive mechanical ventilation. These benefits were consistent across all patient groups studied, including those receiving invasive mechanical ventilation, non-invasive respiratory support, or no respiratory support other than simple oxygen. The benefits of tocilizumab were clearly seen among those also receiving treatment with a systemic corticosteroid, which is now usual standard of care for COVID-19 patients requiring treatment with oxygen.^6^, ^7^

Previous trials have provided some evidence that tocilizumab might shorten time to discharge or reduce progression to invasive mechanical ventilation or death.^9^, ^13^ Since mid-2020, eight randomised, controlled trials of tocilizumab for the treatment of COVID-19 have reported. These include seven small trials (fewer than 100 deaths in each) and the somewhat larger REMAP-CAP trial, which recruited critically ill patients with COVID-19, over 99% of whom required non-invasive respiratory support or invasive mechanical ventilation.^8^, ^9^, ^10^, ^11^, ^12^, ^13^, ^14^, ^15^ Taken together, these previous trials did not show a significant mortality benefit for treatment with tocilizumab (death rate ratio 0·89, 95% CI 0·72–1·11; figure 4). The RECOVERY trial contains around four times as much information as all the previous trials combined. When all nine trials are considered together, allocation to tocilizumab is associated with a significant 14% proportional reduction in 28-day mortality. These results suggest that in COVID-19 patients who are hypoxic and have evidence of systematic inflammation, treatment with a combination of a systemic corticosteroid plus tocilizumab would be expected to reduce mortality by about one-third for patients receiving simple oxygen and nearly one-half for those receiving invasive mechanical ventilation.^6^

The RECOVERY results support the use of tocilizumab. Our results show that the benefits of tocilizumab extend to a broad group of patients receiving oxygen, with or without other forms of respiratory support, and that those benefits include a reduction in the need for invasive mechanical ventilation and renal replacement therapy. Since complicating bacterial infections are infrequent in the early hospitalisation period of COVID-19, this recognised concern in relation to the use of tocilizumab would be lessened with earlier use.^20^ On the basis of the ISARIC4C database, approximately 49% of hospitalised COVID-19 patients in the UK would meet our inclusion criteria and hence would benefit from tocilizumab (ISARIC4C Investigators, personal communication). Sarilumab, an alternative IL-6 antagonist, is available but evidence of its efficacy is inconclusive^15^, ^21^, ^22^ and the results of the largest trial (NCT04315298) are not yet published.

Strengths of this trial included that it was randomised, had a large sample size, and included patients requiring various amounts of respiratory support (from simple oxygen through to invasive mechanical ventilation) and has 99% completeness of follow-up for the primary outcome. CRP was chosen as the biomarker for inflammation in this study since it is widely used and affordable worldwide, it is correlated with serum IL-6 concentrations, and early clinical studies of COVID-19 had reported it to be associated with severity and prognosis, with a value of greater than 50 mg/L associated with severe disease and a concentration of around 75 mg/L distinguishing fatal from non-fatal cases.^23^, ^24^, ^25^, ^26^, ^27^, ^28^ Whether hypoxic patients with a CRP of less than 75 mg/L could benefit from tocilizumab is unknown. There are some limitations. We did not collect detailed information on non-COVID infections. Following random assignment, 16% of patients in the tocilizumab group reportedly did not receive this treatment and the reasons for this were not recorded. The size of the effects of tocilizumab reported in this paper are therefore an underestimate of the true effects of actually using the treatment. Hospital stay is very long for many of these patients and some outcomes beyond 28 days have not yet been captured. The preplanned analyses at 6 months will, however, provide additional information on the full effects of tocilizumab on clinical outcomes. Further work is also needed to consider the health economic benefits of tocilizumab and related IL-6 inhibitors in terms of both patient outcomes and usage of health-care resources (duration of hospital stay, and frequency of invasive mechanical ventilation and renal replacement therapy).

The RECOVERY trial has shown that for patients hospitalised with severe COVID-19, treatment with tocilizumab reduces mortality, increases the chances of successful hospital discharge, and reduces the chances of requiring invasive mechanical ventilation. These benefits are additional to those previously reported for dexamethasone. These findings require an update to clinical guidelines, which has already begun, and efforts to increase the global availability and affordability of tocilizumab.^29^, ^30^

Correspondence to: Prof Peter W Horby and Prof Martin J Landray, RECOVERY Central Coordinating Office, Richard Doll Building, Old Road Campus, Roosevelt Drive, Oxford OX3 7LF, UK recoverytrial@ndph.ox.ac.uk

For **policy and procedures** see https://www.ndph.ox.ac.uk/data-access

## Data sharing

The protocol, consent form, statistical analysis plan, definition and derivation of clinical characteristics and outcomes, training materials, regulatory documents, and other relevant study materials are available online. As described in the protocol, the trial steering committee will facilitate the use of the study data and approval will not be unreasonably withheld. De-identified participant data will be made available to bona fide researchers registered with an appropriate institution within 3 months of publication. However, the steering committee will need to be satisfied that any proposed publication is of high quality, honours the commitments made to the study participants in the consent documentation and ethical approvals, and is compliant with relevant legal and regulatory requirements (eg, relating to data protection and privacy). The steering committee will have the right to review and comment on any draft manuscripts before publication. Data will be made available in line with the policy and procedures. Those wishing to request access should complete the form.

## Declaration of interests

The authors have no conflict of interest or financial relationships relevant to the submitted work to disclose. No form of payment was given to anyone to produce the manuscript. All authors have completed and submitted the International Committee of Medical Journal Editors form for disclosure of potential conflicts of interest. The Nuffield Department of Population Health at the University of Oxford has a staff policy of not accepting honoraria or consultancy fees directly or indirectly from industry.
